# Taxonomic reassessment of *Hydralmosaurus* as *Styxosaurus*: new insights on the elasmosaurid neck evolution throughout the Cretaceous

**DOI:** 10.7717/peerj.1777

**Published:** 2016-03-15

**Authors:** Rodrigo A. Otero

**Affiliations:** Laboratorio de Ontogenia y Filogenia/Departamento de Biología, Facultad de Ciencias, Universidad de Chile, Santiago, Chile

**Keywords:** *Hydralmosaurus*, *Styxosaurus*, Cervical vertebrae morphology, Styxosaurinae elasmosaurid evolution, Cretaceous

## Abstract

Two extremely-long necked elasmosaurids, AMNH 1495, holotype of *Hydralmosaurus serpentinus*, and AMNH 5835, previously referred to *H. serpentinus*, are here reviewed in detail. Unique features of the cervical vertebrae, which are only present on elasmosaurids from the Western Interior Seaway, are recognized based on these specimens and by comparison with penecontemporaneous taxa with biogeographic affinities. Phylogenetic analysis, bivariate graphic analysis of cervical vertebrae proportions, comparisons of different cervical vertebral types, paleobiogeographic distribution and study of the elasmosaurid axial evolution throughout the Cretaceous are here integrated. As a result, at least two separate lineages within the Elasmosauridae are identified by independently acquired extremely-long necks (over 60 cervical vertebrae). First, a still scarcely known lineage is so far represented by the lower Cenomanian *Thalassomedon haningtoni*, the Turonian *Libonectes morgani* and close relatives. A second lineage is here defined as a new clade, the Styxosaurinae, which groups the Campanian genera *Terminonatator*, *Styxosaurus* (=‘*Hydralmosaurus*’), *Albertonectes* and *Elasmosaurus*, the two latter forming a derived branch that includes the most extreme amniote necks known to date (more than 70 cervical vertebrae). Phylogenetic analysis supports AMNH 1495 and AMNH 5835 as being closely related to *Styxosaurus snowii*. Therefore, the species *Styxosaurus browni* is re-validated, while AMNH 1495 is here referred to *Styxosaurus* sp. This research also recognizes the ‘Cimoliasauridae’ (*nomen dubium*) as a paraphyletic group but informative of a plesiomorphic cervical vertebral morphology of elasmosaurids which was persistent throughout the whole Cretaceous and from whom aristonectines, styxosaurines and *Thalassomedon* and close relatives are derived. The genus *Hydralmosaurus* is recommended for being abandoned.

## Introduction

Elasmosaurid plesiosaurians, historically characterized by extremely long necks, are one of the most distinctive Mesozoic marine reptiles (Cope, 1868; Welles, 1943; Carpenter, 1997). This clade was one of the first plesiosaurian groups formalized, mostly based on the remarkable find of ANSP 10081, type of *Elasmosaurus platyurus* Cope, 1868, from the Campanian of the Western Interior Seaway of United States. This animal, unique at the time, possessed 72 cervical centra, with a neck length over 6 m. This specimen was the basis for the clade Elasmosauridae, a taxonomical concept valid to this day, with abundant representatives found during the Cretaceous and distributed worldwide (Vincent et al., 2011).

Further records from the Western Interior Seaway proved the existence of other elasmosaurids with long but likely shorter necks (cervical vertebral counts under 72), giving additional support to the historical distinctive feature of elasmosaurids.

However, plesiosaurians other than the Elasmosauridae independently acquired a high number of cervical centra. Particularly, among Tithonian Arctic cryptoclidids there are representatives with up to 60 cervical vertebrae (Knutsen, Druckenmiller & Hurum, 2012a; Knutsen, Druckenmiller & Hurum, 2012b), showing that an extremely long neck is not an exclusive feature of Elasmosauridae. However, in the past few decades a growing consensus regarding the diagnostic features of the Elasmosauridae has provided a set of both cranial and postcranial characters that allow the distinction of this group from other plesiosaurians, independent of the neck length (Welles, 1952; Welles, 1962; Bardet, Godefroit & Sciau, 1999; Gasparini et al., 2003; Ketchum & Benson, 2010; Benson & Druckenmiller, 2014; Sachs & Kear, 2015). Despite the abundant record of elasmosaurids worldwide, the internal relationships of the clade remain unclear and, although there have been various attempts to clarify them (O’Keefe, 2001; Sato, 2002; Kubo, Mitchell & Henderson, 2012), only one internal clade, the Aristonectinae (Otero, Soto-Acuña & Rubilar-Rogers, 2012), has been distinguished. This internal clade groups several Late Cretaceous derived forms from the Southern Hemisphere and particularly from the Weddellian Biogeographic Province.

This study reviews two historical specimens from the Late Cretaceous of the Western Interior Seaway (AMNH 1495 and AMNH 5835), collected by Edward Drinker Cope in 1876, and by Barnum Brown in 1904, respectively. Although the taxonomical affinities of these specimens have been discussed previously (Cope, 1877; Welles, 1943; Welles, 1952), to date there are only partial osteological descriptions of them and these are mostly restricted to the skull (Carpenter, 1999; Sato, 2002), thus encumbering any postcranial comparison with other elasmosaurids. AMNH 1495 and AMNH 5835, two typical North American elasmosaurids from the Western Interior Seaway, are directly compared with representatives from the Weddellian Biogeographic Province. A junior synonym of the Elasmosauridae, the ‘Cimoliasauridae’, is also reviewed. As a result, both studied specimens from the Western Interior Seaway are taxonomically reassessed. Additional phylogenetic and bivariate analyses allow the recognition of disparate cervical centra among elasmosaurids, represented by very elongate centra, and also by very short centra. Such evolutionary events are contrasted with their biogeographic occurrences, their respective axial formulae, and their associated pectoral girdle changes. As a result, a new clade is here proposed, grouping all the Campanian elasmosaurids from the Western Interior Seaway that possessed extremely long necks. Such adaptation is not present in elasmosaurids outside the Western Interior Seaway and can now be distinguished from other long-necked elasmosaurids by the presence of a singular type of cervical vertebrae. This study also recognizes that elasmosaurids with cervical vertebrae shorter than those of ANSP 10081, which have been considered typical of the ‘Cimoliasauridae’ (DeLair, 1959; O’Keefe, 2001; Smith, 2003), are actually the most common type of cervical centra among elasmosaurids, while very long cervical vertebrae are a disparate event only restricted to the Western Interior Seaway.

## Material and Methods

**Specimens reviewed**—Two elasmosaurid specimens are here reviewed. AMNH 1495, holotype of *Hydralmosaurus serpentinus* (Cope, 1877), which comprises a fairly complete axial skeleton, incomplete pectoral and pelvic girdles, and both forelimbs. AMNH 5835, holotype of *Styxosaurus browni* Welles, 1952, later referred to *H. serpentinus* (*sensu* Carpenter, 1999). This comprises the skull, complete neck, pectoral girdle, left forelimb and anterior trunk elements.

Cervical vertebrae of AMNH 1495 have been historically numbered starting with c3. This was deliberately done for indicating the absence of two anterior centra, as is suggested by Welles (1943) in its first description. On the other hand, cervical vertebrae of AMNH 5835 have been numbered starting on c1. In order to minimize confusion, this research used the original numbering proposed by Welles (1943) to refer to each individual centra.

**Phylogenetic analysis**—Benson & Druckenmiller (2014) constructed a phylogenetic datamatrix of 270 unordered morphological characters, and 80 operational taxonomic units (OTU). The pistosaurian *Yunguisaurus liae* was established as the outgroup. Twelve new OTUs were added (Table 1), represented by seven elasmosaurids from the Western Interior Seaway, one from the Pacific of North America, and five elasmosaurids from the Weddellian Biogeographic Province. A new data row for *Aristonectes parvidens* (holotype, MLP 40-XI-14-6) and for *Kaiwhekea katiki* (OU 12649) were also included based on personal review of each specimen. For AMNH 1495, scoring of the frontlimb characters was based on Welles (1943) and Welles (1952) because the frontlimb is currently lost. Additional character states are listed in Table 2. Analysis was performed with TNT software (Goloboff, Farris & Nixon, 2008). Bootstrap resampling was performed with 2,000 replicates in all cases (Standard, New Tech Search, tree fusing), to test the stability of the cladograms. For intermediate to large datasets as is the current case (105 OTUs; 270 characters), the calculation of Bremer Support can be problematic because support values can turn out to be severe overestimations of support (Bremer, 1994; Müller, 2005). Thus, usage of Bremer Support was precluded.

**Table 1:** List of new OTUs. Operational taxonomical units added to the datamatrix of Benson & Druckenmiller (2014) for phylogenetic analysis.

OTU	Collection number	Age	Stratigraphic provenance	Locality	References	Province	Graphic bivariate analysis
*Thalassomedon haningtoni*	DMNH 1588	Lower Cenomanian	Graneros Shale	Colorado, USA	Welles, 1943	WIS	x
*Elasmosaurus platyurus*	ANSP 10081	Lower Campanian	Pierre Shale Group	Kansas, USA	Cope, 1868; Cope, 1869; Sachs, 2005	WIS	x
*Styxosaurus snowii*	KUVP 1301	Lower Campanian	Niobrara Formation	Kansas, USA	Williston, 1890; Welles, 1952; Carpenter, 1999	WIS	
*Styxosaurus* sp.	AMNH 1495	Middle Campanian	Pierre Shale Group, Sharon Springs Formation	Nebraska, USA	Cope, 1877; Welles, 1943	WIS	x
*Styxosaurus browni*	AMNH 5835	Middle to upper Campanian	Pierre Shale Group, Sharon Springs Formation	South Dakota, USA	Welles, 1952	WIS	x
*Terminonatator pointeixensis*	RSM P2414.1	Upper Campanian	Bearpaw Formation	Pointeix, Canada	Sato, 2003	WIS	
*Albertonectes vanderveldei*	TMP 2007.011.0001	Middle to upper Campanian	Bearpaw Formation	Lethbridge, Canada	Kubo, Mitchell & Henderson, 2012	WIS	
*Mauisaurus haasti*	CM Zfr 115	Upper Campanian	Conway Formation	Jed River, New Zealand	Hiller et al., 2005	WBP	x
*Tuarangisaurus keyesi*	GNS CD 425	Upper Campanian–lower Maastrichtian	?	Mangahouanga Stream, New Zealand	Wiffen & Moisley, 1986	WBP	
*Alexandronectes zealandiensis*	CM Zfr 73 + 91	Lower Maastrichtian	Conway Formation	Waipara River, New Zealand	Hiller & Mannering, 2004; Otero et al., 2016	WBP	
*Morturneria seymourensis*	TTU P 9219	Upper Maastrichtian	López de Bertodano Formation	Seymour Island, Antarctica	Chatterjee & Small, 1989	WBP	x
*Aristonectes quiriquinensis*	SGO.PV.957	Upper Maastrichtian	Quiriquina Formation	Central Chile	Otero et al., 2014c	WBP	x

**Table 2:** Modifications introduced to the datamatrix. New characters and states introduced to the datamatrix of Benson & Druckenmiller (2014).

Character number	Original description (Benson & Druckenmiller, 2014)	Modifications	References
8	Inclination of the suspensorium: sub-vertical or weakly inclined (∼80–90°) (0); significantly inclined (<70°) (1).	New state: (2) suspensorium absent, squamosals joins the pterygoids and parietals	Carpenter (1999, character 9), Sato (2002, character 75), Druckenmiller & Russell (2008, character 36), Ketchum & Benson (2010, character 45).
49	Inter-squamosal suture along the dorsal midline in lateral view: low and rounded (0); raised ∼1/3 orbit height dorsally relative to skull table (1); raised abruptly and substantially dorsally relative to skull table (2).	New state: (3) squamosals joins the parietals	State 2 taken from Benson et al. (2012b, character 43).
53	Squamosal arch, posterior margin in dorsal view: dorsal processes extend anterolaterally (0); approximately straight, squamosal dorsal processes extend laterally from midline contact (1); V-shaped, squamosal dorsal processes extend posterolaterally (2).	New state: (3) squamosals do not meet dorsomedially	Benson & Druckenmiller (2014).
70	Opisthotic, paraoccipital process length relative to height of exoccipital body: subequal (0); long, at least 1.3 times as long as body height (1).	New state: (2) over 3 times the height of the exoccipital-opisthotic	Benson, Evans & Druckenmiller (2012a, character 53); Modified from O’Keefe (2001, character 46), Sato (2002, character 64), O’Keefe & Wahl (2003, character 24), Großmann (2007, character 21), Druckenmiller & Russell (2008, character 61), Smith & Dyke (2008, character 49).
71	Opisthotic, orientation of paraoccipital process relative to ventral surface of exoccipital in posterior view: inclined dorsally (0); paraoccipital process oriented parallel to ventral surface of exoccipital (1); inclined ventrally (2).	New state: (3) posteriorly straight	Benson, Evans & Druckenmiller (2012a, character 54); Modified from O’Keefe (2001, character 48), Sato (2002, character 67), O’Keefe & Wahl (2003, character 26), Großmann (2007, character 22), Druckenmiller & Russell (2008, character 65), O’Keefe & Street (2009, character 22), Ketchum & Benson (2010, character 77).
72	Opisthotic, morphology of articulation with suspensorium: anterior surface of expanded lateral end makes broad contact with suspensorium (0); lateral end unexpanded, lateral/terminal surface makes narrow contact with suspensorium (1).	New state: (2) long contact along half of the lateromedial margin of the paraoccipital process	Benson, Evans & Druckenmiller (2012a, character 55); Modified from O’Keefe (2001, character 49), O’Keefe & Wahl (2003, character 27).
73	Opisthotic, shaft of paraoccipital process cross section: subcircular, dorsoventral height subequal to anteroposterior width (0); dorsoventrally flattened; anteroposterior width much greater than dorsoventral height (1).	New state: (2) proximally subcircular and distally flattened	Benson, Evans & Druckenmiller (2012a, character 56).
86	Parasphenoid, ventral surface anteriorly: covered by pterygoids anterior to the posterior interpterygoid vacuities (0); visible through V-shaped notch in posterior pterygoid contact anterior to posterior interpterygoid vacuities (1).	New state: (2) parasphenoid extended broad and anterior to the posterior margin of the interpterygoid vacuities through a medial projection	Benson, Evans & Druckenmiller (2012a, character 65).
109	Ectopterygoid/pterygoid boss/flange: absent (0); ventrally deflected posterior margin forms flange (1); rugose ventral boss present (2).	New state: (3) ectopterygoid forms a flange mostly with palatine and has a scarce contact with pterygoid	Benson, Evans & Druckenmiller (2012a, character 84); Modified from O’Keefe (2001, character 84), Sato (2002, character 57), Druckenmiller & Russell (2008, character 47), Ketchum & Benson (2010, character 58) by interposition of state 1 from Sato (2002, character 56).
163	Cervical ribs, size and orientation of distal processes: marked anterior and posterior processes throughout cervical rib series, combined long axis of processes oriented approximately anteroposteriorly (0); processes reduced, especially anterior process, combined long axis oriented posteroventrally (1); large, anteroposteriorly expansive, sheet-like ribs with prominent processes (2).	New state: (3) cervical ribs without anterior process but inflected rostrally	O’Keefe (2001, character 123), Sato (2002, character 146), O’Keefe & Wahl (2003, character 68), Druckenmiller & Russell (2008, character 115), Smith & Dyke (2008, character 71), O’Keefe & Street (2009, character 59), Ketchum & Benson (2010, character 134), Vincent et al. (2011, character 52), Benson, Evans & Druckenmiller (2012a, character 124).
189	Caudal centra, outline of middle caudal centra in anterior view: suboval (0); subrectangular, chevron facets widely spaced and located ventrolaterally, ventral surface approximately flat giving a subrectangular appearance to centrum in anterior view (1).	New state: (2) octagonal outline due large facets fo the neural arch and ribs, plus presence of a flattened ventral surface	Benson, Evans & Druckenmiller (2012a, character 150).

**Bivariate analysis**—Cervical vertebral indices used follow the definitions by Welles (1952): height/length ratio (*HI* = 100∗*H*∕*L*); breadth/length ratio (*BI* = 100∗*B*∕*L*); rate of vertebral elongation (*VLI* = 100∗*L*∕(0.5∗(*H* + *B*))). Graphic usage of these indices follows the methodology of O’Gorman, Gasparini & Salgado (2013). Specimens considered for the bivariate analysis are listed on Table 3. This analysis was intended to evaluate the presence of disparate cervical centra among elasmosaurids. VLI indices of the three main groups obtained in the bivariate analysis were statistically tested. VLI of aristonectines consider 39 available values; styxosaurines, 186 values; other non-aristonectine and non-styxosaurine elasmosaurids, 206 available values. Because the sample sizes are unequal, a non-parametric analysis was performed. For this case, a Kruskal-Wallis test is considered, because the assumption is that the samples have unequal sizes. The Kruskal–Wallis Test was performed with the PAST software V.1.95 (Hammer, Harper & Ryan, 2001). A *p*-value of 0.01 was considered significant.

**Table 3:** Additional elasmosaurid specimens considered for the bivariate graphic analysis. List of taxa with cervical measurements used on the graphic bivariate analysis, indicating their respective locality, horizon and age.

OTU	Collection number	Age	Stratigraphic provenance	Locality	References	Province
Elasmosauridae indet.	SGO.PV.6506	Middle Maastrichtian	Quiriquina Formation	Central Chile	Otero et al., 2014a	WBP
*Hydrotherosaurus alexandrae*	UCMP 33912	Maastrichtian	Moreno Formation	Fresno, California, USA	Welles, 1943	WIS
*Callawayasaurus colombiensis*	UCMP 38349	Lower Aptian	Paja Formation	Boyacá, Colombia	Welles, 1962	Putumayo Basin
‘*Alzadasaurus tropicus*’	AMNH 6796	Cenomanian-Turonian	Querecual Limestone	Orituco, Venezuela	Colbert, 1949	Putumayo Basin
‘*Cimoliasaurus magnus*’	AMNH 2554	Maastrichtian	Hornerstown Formation	New Jersey, USA	Leidy, 1851	North Atlantic
*Kaiwhekea katiki*	OU 16449	Lower Maastrichtian	Katiki Formation	Shag Point, New Zealand	Cruickshank & Fordyce, 2002	WBP
*Libonectes morgani*	SMUSMP 69120	Turonian	Britton Formation	Dallas, USA	Welles, 1949; Carpenter, 1999	WIS
*Futabasaurus suzukii*	NSM PV15025	Santonian	Tamayama Formation	Futaba, Japan	Sato, Hasegawa & Manabe, 2006	North Pacific

**Neck length estimation**—For those specimens here reviewed which preserve fairly complete necks, an estimation of their effective neck length was calculated. This takes in account the sum of the length of each cervical vertebra. For absent cervical elements or else, for those cervical elements that cannot be measured, an average value is calculated based on the sum of every centrum length, divided by the number of cervical centra. This value was replaced on each missing or unavailable element and it was considered in the total cervical length sum.

**Nomenclatural acts**—The electronic version of this article in Portable Document Format (PDF) will represent a published work according to the International Commission on Zoological Nomenclature (ICZN), and hence the new names contained in the electronic version are effectively published under that Code from the electronic edition alone. This published work and the nomenclatural acts it contains have been registered in ZooBank, the online registration system for the ICZN. The ZooBank LSIDs (Life Science Identifiers) can be resolved and the associated information viewed through any standard web browser by appending the LSID to the prefix “http://zoobank.org/”. The LSID for this publication is: urn:lsid:zoobank.org:pub:1E0DA2E1-50A9-453D-BC3F-D036DA44B389. The online version of this work is archived and available from the following digital repositories: PeerJ, PubMed Central and CLOCKSS.

## Localities, Horizon and Ages of the Re-described Specimens

**Figure 1:**
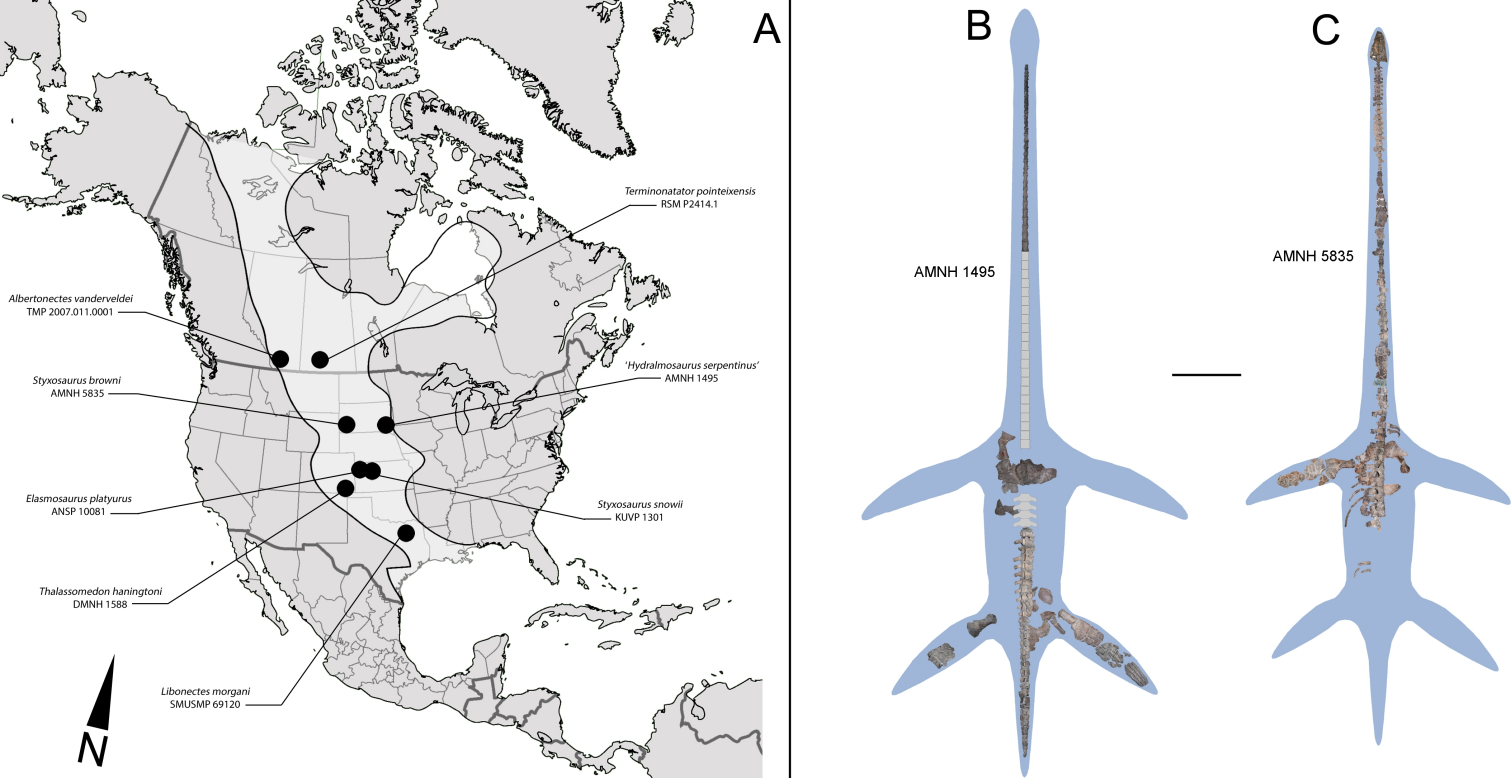
North American localities with elasmosaurid finds and elasmosaurid specimens here reviewed. (A) ‘Mid’ and Late Cretaceous elasmosaurid localities from United States and Canada. An estimated outline of the Western Interior Seaway (WIS) is shown on light grey. (B) Composite and estimated outline of AMNH 1495 from the WIS, here reviewed. (C) Composite and estimated outline of AMNH 5385 from the WIS, here reviewed. Scale bar equals 1 m.

The respective localities, horizons and ages of additional specimens considered on this study are summarized in Table 1. Localities of elasmosaurids from the Western Interior Seaway are indicated in Fig. 1A. Particularly, the specimens studied here are:

**AMNH 1495** (Fig. 1B)—Locality of provenance was regarded by Cope (1877) as from “the blue shale of Cretaceous N°3, in a bluff of Nebraska, on the southwest side of the Missouri, between Sioux City, Iowa, and Yankton, Dakota”. Welles (1943) indicates an equivalence for Cretaceous N°3 as Niobrara or Pierre Shale.

**AMNH 5835** (Fig. 1C)—Locality of provenance originally indicated by Welles (1952) is Mule Creek, 15 miles west of Edgemont, South Dakota, USA. The specimen was collected in 1904 by Barnum Brown, placing its horizon as Niobrara. Additional comments by Welles (1962) about the lithology observed on the hosting blocks correlate them with the Pierre Shale Group.

The lithology associated to AMNH 1495 and AMNH 5835 is consistent with that described for the ‘Sharon Springs Member’ (Elias, 1931). The latter unit was later re-ranked to formation level within the Pierre Shale Group. The Sharon Springs Formation comprises black to gray, highly organic claystone with a fissile parting, commonly with concretions and numerous yellow-weathered bentonite beds, particularly near the base (Martin, Bertog & Parris, 2007). The age of the Sharon Springs Formation is constrained by ammonoid biozones, particularly in the range of *Baculites obtusus* through *Baculites perplexus*. This indicates a middle Campanian age (Cobban, 1993; Obradovich, 1993; Grandstein et al., 1994; Bertog, 2010).

## Internal Relationships of the Elasmosauridae: Taxonomical Hypothesis

As previously expressed by O’Keefe & Hiller (2006), “variability is the rule” on the elasmosaurid neck. These authors noted the existence of taxa with conservative necks, calling them non-elongate taxa, and a second group, denominated as elongate taxa, possessing very long centra in the mid-cervical region. Also, at least three sources of differences on cervical vertebrae were detected: ontogenetic allometry, intracolumn variation and taxonomic variation. Later, Otero et al. (2015a) proposed an informal segregation of three groups within Elasmosauridae. In addition to the clade Aristonectinae (Otero, Soto-Acuña & Rubilar-Rogers, 2012), these authors separated those forms from North America, possessing extremely-long necks, from a second elasmosaurid type with comparatively shorter centra (but longer than those of aristonectines). The latter were considered basal representatives, and therefore, informally called ‘plesiomorphic forms’. Elasmosaurids with this type of cervical vertebrae precisely match the classic concept of ‘Cimoliasauridae’ DeLair, 1959 (*nomen dubium*), currently considered as junior synonym of Elasmosauridae (O’Keefe & Street, 2009). The polyphyletic status of ‘Cimoliasauridae’ and its broad usage as a “waste-basket” taxon are not subject to discussion. However, information exposed in this research shows that these type of cervical vertebrae can be taxonomically informative, although they cannot resolve to genus nor species level. In order to avoid direct homologation of this cervical type neither with the taxon ‘Cimoliasauridae’, nor with the genus ‘*Cimoliasaurus*’ Leidy, 1851, the ‘*Cimoliasaurus*’-grade cervical morphotype is here proposed. This definition reinstates the cervical features orginally described by Leidy (1851) for the type specimen of ‘*Cimoliasaurus magnus*’ (*nomen dubium*), currently under the acronym and numeration AMNH 2554. Cervical centra of AMNH 2554 are distinguishable intermediates between the elongated cervical centra present in elasmosaurids from the Western Interior Seaway such as *Elasmosaurus platyurus*, *Styxosaurus snowii* (Williston, 1890), ‘*Hydralmosaurus serpentinus*’ (Cope, 1877), and *Terminonatator pointeixensis* Sato, 2003, and those axially shortened centra of aristonectines such *Aristonectes parvidens* Cabrera, 1941, *Aristonectes quiriquinensis* Otero et al., 2014c, *Morturneria seymourensis* (Chatterjee & Small, 1989) and *Kaiwhekea katiki* Cruickshank & Fordyce, 2002. The relationships between these three morphotypes are among the subjects of study of this research.

## Systematic Paleontology

An emended diagnosis of the clade Elasmosauridae is first proposed based on the information previously presented.

**Table:** 

DIAPSIDA Osborn, 1903
SAUROPTERYGIA Owen, 1860
PLESIOSAURIA De Blainville, 1835
PLESIOSAUROIDEA Welles, 1943
XENOPSARIA Benson & Druckenmiller, 2014
ELASMOSAURIDAE Cope, 1869

**Type Species**—ANSP 10081, holotype of *Elasmosaurus platyurus* Cope, 1868. Logan County, Kansas, USA. Lower Pierre Shale Group, lower–middle Campanian.

**Diagnosis**—Xenopsarian plesiosaurians mostly restricted to the Cretaceous, unambiguously distinguished by the following combination of characters: cervical vertebrae with ventral notch (shallow in basal elasmosaurids and well-marked in Late Cretaceous forms) giving them a bilobed articular outline; neural arches much narrower than their respective centra; pre- and postzygapophyses as narrow as the neural arch, dorsally recurved and partially meeting in the midline; medial embayment of the posterior portion of the coracoid. A plesiomorphic number of cervical vertebrae higher than 40 is a feature present in all known elasmosaurids preserving complete enough necks. However, this feature is shared with derived cryptoclidids such as *Djupedalia* and *Spitrasaurus* (Knutsen, Druckenmiller & Hurum, 2012a; Knutsen, Druckenmiller & Hurum, 2012b). Derived Late Cretaceous forms within Elasmosauridae include the largest necks among sauropterygians (and among amniotes), with over 70 cervical vertebrae, unique axially elongated centra and skulls reduced in length. Late Cretaceous elasmosaurids also include atavic forms retaining ca. 45 cervical vertebrae with shortened centra and enlarged skulls (aristonectines).

**Table:** 

STYXOSAURINAE new clade

**Type species**—AMNH 5385, *Styxosaurus* (=‘*Hydralmosaurus*’) *browni* Welles, 1952.

**Etymology**—Following the genus *Styxosaurus* Welles, 1943, which includes the type species.

**Diagnosis**—Clade of Campanian elasmosaurids from the Western Interior Seaway, distinguished by the following combination of characters: 60 or more cervical vertebrae (the minimal number of cervical vertebrae is based on the count of AMHN 1495 which has 58 cervical vertebrae and few estimated missing cervical centra); presence of mid cervical centra axially elongated reaching a length between two thirds to twice its width, being as broad as high (“can-shaped” cervical vertebrae); neck much longer than the trunk; skull less than one tenth the cervical series length; plesiomorphic number of 17–19 dorsal vertebrae (see ‘Discussion’).

**Phylogenetic definition**—The Styxosaurinae includes the genera *Terminonatator*, *Styxosaurus* (=‘*Hydralmosaurus*’, below), *Albertonectes*, *Elasmosaurus*, their most recent ancestor and all descendants.

**Table:** 

Genus *HYDRALMOSAURUS* Welles, 1943, *nomen dubium*

**Type species**—AMNH 1495. *Elasmosaurus serpentinus* (Cope, 1877).

**Original generic diagnosis**—Welles (1943) coined the genus *Hydralmosaurus* considering as diagnostic features: the absence of pectoral and pelvic bars, the absence of lateral longitudinal ridges on posterior cervical vertebrae, the presence of cordiform fenestra on the coracoids, pubes with a concave anterior border, humerus head well-separated from the tuberosity, and a well-developed epipodial foramen.

**Comments**—With the exception of the concave anterior pubis border, all these characters are ambiguous because they can be found in other elasmosaurids. In addition, a few of these characters even depend on the ontogenetic stage of the specimen (see ‘Discussion’). Carpenter (1999) indicates a separation of *Hydralmosaurus* from *Libonectes*, as well as from *Styxosaurus* and *Thalassomedon*, based on the presence of 62 cervical vertebrae of the first, and 63 cervical vertebrae on the two latter taxa. This feature is arguable because it assumes an excellent preservation as well as a rigorous recovery of the complete neck. Considering that all these specimens were collected during the late 19th century, with documented cases of discrepancy on the real cervical number in some specimens (Everhart, 2005; Sachs, 2005; Sachs, Kear & Everhart, 2013), this putative character should be rejected. This study cannot verify the condition of the pubis with an anterior concave margin, because this is fragmentary and preserved on several pieces without contact between them. An additional diagnostic feature described by Carpenter (1999) is the humerus with a pronounced posterior expansion on its distal end, unlike all other elasmosaurids, and the absence of pectoral and pelvic bars. AMNH 1495 does not preserve its humeri.

Summarizing, the morphologic features of AMNH 1495, so far considered as diagnostic to genus level, should be rejected. New diagnostic features need to be identified for a taxonomical reassessment of this specimen.

**Remarks**—A propodial referred as the humerus of AMNH 1495 was presented by Carpenter (1999: Fig. 7A) through a drawing taken from Welles (1952: Fig. 4B). The first description of *H. serpentinus*, Welles (1943: Fig. 29) exhibited the pectoral girdle and one forelimb. This current review of AMNH 1495 allows recognizing that the pectoral outline is consistent with the preserved portions of this specimen. Nonetheless, the two preserved propodials do not match the outlines nor the preserved portions described by Welles (1943) and Welles (1952). Interestingly, the original forelimb outline described by Welles (1943: Fig. 29) and Welles (1952: Fig. 4B) and subsequently cited by Carpenter (1999: Fig. 7A) precisely matches the forelimb of AMNH 5835 (type of *Styxosaurus browni* Welles, 1943, below), both in shape and in the preserved portions. This was illustrated by Welles (1952: Fig. 7). AMNH 5835 forelimb was drawn from a dorsal view, while the putative forelimb of AMNH 1495 (Welles, 1952: Fig. 4B) was drawn from a ventral view. Thus, it is likely that Welles confused the forelimbs of both specimens, with the subsequent taxonomical consequences. After these publications, no further description of the AMNH 1495 forelimb has been provided. Furthermore, among the material, the forelimb of AMNH 1495 was not found.

Due to the unsatisfactory status of AMNH 1495, which is the only specimen fixed to *Hydralmosaurus*, a reassessment is proposed (see below).

**Table:** 

Genus *STYXOSAURUS* Welles, 1943

**Type species**—*Cimoliosaurus* (*Elasmosaurus*?) *snowii* Williston, 1890. Skull and 28 cervical vertebrae. Currently under the collection number KUVP 1301.

**Locality, horizon and age**—South Dakota, USA. Niobrara Formation, middle-to-upper Campanian.

**New referred specimens**—AMNH 5835: Skull, complete neck, anterior trunk and left forelimb. AMNH 1495: Fairly complete axial skeleton, partial girdles, right and left hindlimbs.

**Locality, horizon and age**—AMNH 5835: Mule Creek, 15 miles west of Edgemont, South Dakota. AMNH 1495: southwest side of the Missouri, between Sioux City, Iowa, and Yankton, Dakota. Both specimens are from the Sharon Springs Formation, lower Pierre Shale Group. *Baculites obtusus* - *Baculites perplexus* biozone, middle Campanian.

**Previous referred specimens**—Six additional specimens have been previously referred to this genus by Carpenter (1999): KUVP 434, holotype of ‘*Thalassiosaurus ischiadicus*’ Welles, 1943 (*nomen dubium*); USNM 11910, posterior cervicals, dorsal, sacral and caudal vertebrae, and pelvis (Carpenter, 1999); YPM 1130, holotype of ‘*Alzadasaurus kansasensis*’ (Welles, 1952) (*nomen dubium*); YPM 1644, a partial axial skeleton, pectoral girdle, pubis and humerus; and YPM 1645, holotype of ‘*Thalassonomosaurus marshii*’ (Williston, 1903) (*nomen dibium*). All of them comprise incomplete postcranial skeletons from the lower Campanian of the Niobrara Formation. Finally, SDSM 451, holotype of ‘*Alzadasaurus pembertoni*’ (Welles & Bump, 1949) is a fairly complete skeleton from the lower Campanian of the Pierre Shale.

KUVP 434: For this specimen, Williston (1903) indicates the presence of both ischia, ilia, seven cervical vertebrae and an undetermined number of caudal vertebrae. Later, Williston (1906) emended the taxon to ‘*Elasmosaurus ischiadicus*’, likely based on the features of the cervical vertebrae. Welles (1943) referred this specimen to a new genus, ‘*Thalassiosaurus ischiadicus*’, distinguishing it from the genus *Elasmosaurus* (fixed to ANSP 10081) by having a “pubis convex anteriorly as in *E. platyurus*, but without median bar”. The pelvic girdle of ANSP 10081 is lost, making any comparison impossible. Moreover, pelvic features are not diagnostic enough for a genus-level determination. Welles (1943) also added some features of the hind limb to the diagnosis, however, this portion is not preserved on KUVP 434 so the features were based on a second specimen (YPM 1130, see below) referred to the same species by Williston (1906). The anatomic portions of KUVP 434 are not diagnostic to genus-level and should be referred to Elasmosauridae indet.

USNM 11910: It comprises the posterior cervical, dorsal, sacral and caudal vertebrae, plus the pelvis. It was referred by Carpenter (1999) to *Styxosaurus snowii*.

YPM 1130: This specimen comprises both pubes, ischia, one ilium and one limb. It was tentatively referred by Williston (1906) to ‘*Elasmosaurus ischiadicus*’ based on its cervical vertebrae as well as its short ischium. This was later considered as a referred specimen of ‘*Thalassiosaurus ischiadicus*’ by Welles (1943). Later, Welles (1952) considered it as a new genus and species, ‘*Alzadasaurus kansasensis*’ (*nomen dubium*), indicating the presence of 28 cervical vertebrae, 5 pectorals, 3 dorsals, 5 sacrals, 22 caudals, and the right hindlimb (first considered as a forelimb by Williston, 1906). This completeness could be informative for recognizing a congenerity with AMHN 1945 and AMNH 5835, here studied.

YPM 1644: First described by Williston (1906) and referred to ‘*Elasmosaurus*’ *snowii*. It comprises a partial vertebral column (cervical and dorsal vertebrae), pectoral girdles, humerus and pubis. The anatomical identity of the humerus was discussed by Welles (1943), who considered it a femur. If so, this propodial matches the femur of AMNH 1495 and it likely belongs to *Styxosaurus*. Carpenter (1999) considered this specimen a junior synonym of *Styxosaurus snowii*.

YPM 1645: This specimen comprises 32 vertebrae, a scapula, and a nearly complete limb. It was first described by Williston (1906) and referred to *Elasmosaurus* (?) *marshii*. Later, Welles (1943) reasigned it to a new genus, ‘*Thalassonomosaurus*’ *marshii*. The scapula is slightly similar to those of AMNH 5835; the humerus is also very similar to that of AMNH 5835. It likely belongs to *Styxosaurus*.

SDSM 451: This specimen is a nearly complete skeleton referred to ‘*Alzadasaurus pembertoni*’ by Welles & Bump (1949). The skull length (37.5 cm) and general shape are very alike to KUVP 1301. The 61 cervical vertebrae (59 plus the atlas-axis) are remarkably similar and almost match in number to those of AMNH 1495 and AMNH 5835. Furthermore, the scapulae are similar to those of AMNH 5835 (poorly known in AMNH 1495). The coracoids of SDSM 451 and AMNH 5835 have different posterior embayments, however, the re-joining observed on SDSM 451 likely represents a later ontogenetic stage. Finally, the ischia of SDSM 451 are almost identical to those of AMNH 1495, while the ilia are very similar. All these features strongly support SDSM 451 as within the genus *Styxosaurus*.

**Synonyms**—The monospecific genus *Hydralmosaurus* is represented by its type species *H. serpentinus* (AMNH 1495) and by the unique referred specimen AMNH 5835. Both are here referred to the genus *Styxosaurus*, leaving *Hydralmosaurus* as a void taxon. *Hydralmosaurus* is subsequently considered as junior synonymy of *Styxosaurus*.

**Revised generic diagnosis**—Skulls of KUVP 1301 and AMNH 5835 share common features of non-aristonectine elasmosaurids: orbit placed near the half of the skull length; orbit length equivalent to half the length of the temporal fenestra; orbit with reniform ventral margin; postorbital with triangular outline; sigmoidal tooth row with quadrate articulation projected ventrally with respect to the rest of the skull (tooth row higher than the glenoid); presence of caniniform teeth; less than 20 maxillary teeth; squamosals possessing a posteriorly projected boss.

**Differential Diagnosis**—*Styxosaurus* differs from other elasmosaurids in the following cranial characters: 4–5 premaxillary teeth, differing from *Eromangasaurus carinognathus* (7) and from *Elasmosaurus platyurus* (6); 15 maxillary teeth on *Styxosaurus*, differing from *Callawayasaurus colombiensis* (17+), *Hydrotherosaurus alexandrae* (9), *Thalassomedon haningtoni* (7), *Terminonatator pointeixensis* (13) and *Zarafasaurus oceanis* (10–11). The genus *Styxosaurus* has a number of premaxillary and maxillary teeth similar to *Libonectes morgani*, *Tuarangisaurus keyesi*, *Futabasaurus suzukii* (maxillary count unknown), although, it differs from *L. morgani* in having a less axially elongated premaxilla, as well as a smaller and not rounded orbit. *Styxosaurus* also differs from *T. keyesi*. The latter posesses an orbit comparatively larger, with a craniocaudal length close to that of its temporal fenestra. It also has a prominent dorsal margin of the orbit, which is absent on *Styxosaurus*. Furthermore, *Futabasaurus* has a flat ventral margin of the orbit, contrary to the convexity of the same margin in *Styxosaurus.* In addition, the skulls of KUVP 1301 and AMNH 5835 possess a ridge in the margin of the temporal fossa which is not present in any of the afore mentioned genera and species preserving the temporal bar. Additional postcranial characters rely on AMNH 5835: more than 60 and less than 65 cervical vertebrae; “can-shaped” mid cervical vertebrae with lateral keels; coracoids with an embayment in the posterior midline; humerus with an expanded postaxial distal margin. AMNH 1495 cervical measurements have a remarkable degree of morphological overlap with those of KUVP 1301, except in c3 and c4. However, such differences could be due to the fact that the amount of anterior cervical vertebrae missing in AMNH 1495 is unclear (Welles, 1952: p. 62). Otherwise, based on the cervical values provided by Welles (1952: Table 5), c6 of KUVP 1301 has proportions similar to c7 and c8 of AMNH 1495; c9 of KUVP 1301 and c9 of AMNH 1495 have very similar proportions; c14 of KUVP 1301 has measurements close to c15 of AMNH 1495; and last, c20 of KUVP 1301 is remarkably similar in measurements to c20 or c21 of AMNH 1495. Furthermore, cervical measurements of KUVP 1301 are remarkably similar to those of AMNH 5835, supporting that those three specimens belong to closely related animals. Measurements of the three specimens are summarized in Table 4.

**Table 4:** Cervical measurements of the specimens referred to the genus *Styxosaurus* and measurements of ‘*Cimoliasaurus magnus*’. Measurements of AMNH 1495, AMNH 5835 and AMNH 2554 are presented. Measurements of KUVP 1301 are also provided (taken from Welles, 1952). Estimated values are indicated with an asterisk.

Correlative count	Numeration on specimen	Length	Height	Breadth	Correlative count	Numeration on specimen	Length	Height	Breadth
**AMNH 1495**					**AMNH 5835**				
1	3	39.67	35.15	46.25	1	2	38.46	29.77	40.86
2	4	41.42	34.78	45.60	2	3	41.93	28.36	40.18
3	5	44.09	33.91	48.59	3	4	45.42	33.65	37.51
4	6	44.84	37.08	50.04	4	5	43.38	31.81	39.81
5	7	45.76	38.24	50.43	5	6	49.56	32.77	39.72
6	8	46.77	36.89	50.71	6	7	49.07	33.26	43.07
7	9	53.12	41.65	51.06	7	8	51.05	33.01	46.09
8	10	51.25	38.62	50.27	8	9	53.91	30.55	47.51
9	11	55.87	42.05	51.89	9	10	56.86	34.31	48.07
10	12	56.78	40.65	55.24	10	11	58.05	32.23	51.58
11	13	60.91	42.28	55*	11	12	60.68	33.68	49.47
12	14	56.96	39.52	57.46	12	13	62.33	35.71	51.45
13	15	63.59	45.78	60.44	13	14	64.36	37.55	53.57
14	16	70.44	44.98	62.83	14	15	60.66	37.65	55.32
15	17	73.90	50.48	60.04	15	16	66.04	40.90	53.78
16	18	71.73	54.19	63.18	16	17	68.47	37.44	60.23
17	19	74.31	54.66	64.18	17	18	67.99	38.41	57.48
18	20	79.03	56.84	65.77	18	19	68.63	42.63	60.98
19	21	78.49	56.92	66.07	19	20	70.97	42*	55.86
20	22	80.79	59.64	64.54	20	21	71.06	38.81	67.38
21	23	85.27	60.28	66.57	21	22	72.54	46.42	65.45
22	24	88.07	61.01	69.25	22	23	79.28	–	63.03
23	25	88.21	61.72	71.69	23	24	80.13	50.43	62.94
24	26	92.70	62.39	71.32	24	25			
25	27	94.09	63.75	76.98	25	26	83*	–	–
26	28	96.35	65.72	76.10	26	27	87.35	–	83.43
27	29	97.68	65.78	76.12	27	28	89.38	62*	76.15
28	30	99.05	66.70	77.33	28	29	95.02	62*	84*
29	31	100.42	69.20	80.18	29	30	93.71	69*	–
30	32	100.98	72.81	85.14	30	31	90.34	63.70	–
31	33	104.43	74.37	85.19	31	32	94.05	61.82	–
32	34	108.82	75.07	89.50	32	33	99.85	71.67	–
33	35	88.63	83.17	93.52	33	34	–	–	–
34	36	108.55	80.52	100.83	34	35	104.91	–	85.89
35	37	111.27	79.18	110.08	35	36	102.85	67*	88.51
36	38	112.7	87.89	109.96	36	37	98.23	63.85	92*
37	39	112.55	93*	113.02	37	38	102.29	61.62	99.62
38	40	113.78	97*	112*	38	39	106.60	–	86.24
39	41	107.07	81.85	114.25	39	40	106.71	66.49	104*
40	42	–	–	–	40	41	114.68	70*	102*
41	43	–	–	–	41	42	111.32	69.19	93.07
42	44	111.9	93.43	119.19	42	43	116.82	78.66	99.97
43	45	117.66	97*	111.48	43	44	115.98	79.40	100*
44	46	132.46	99*	112.9	44	45	115.76	78.92	105.97
45	47	115.18	94.42	116.65	45	46	118.48	71.42	108.82
46	48	111.83	101.5	123.86	46	47	116.75	72.24	111.12
47	49	112.79	?	123.71	47	48	113.58	78.49	107.25
48	50	127.3	94.17	126.63	48	49	117.42	78.98	116.98
49	51	108.4	93.24	118.62	49	50	115.85	78.51	119.52
50	52	112.08	–	126.68	50	51	115.69	81.62	126.23
51	53	120.24	88.85	133.6	51	52	118.62	80*	125*
52	54	105.57	89.03	123.82	52	53	118.64	–	112.91
53	55	123.28	95.96	126.32	53	54	115.00	83.84	132.39
54	56	107.87	93.3	122.23	54	55	115.75	84.42	127.90
55	s/n	–	101.14	12.22	55	56	111.33	84.38	132.81
56	s/n	–	–	–	56	57	107.78	90.83	138.64
57	s/n	–	–	–	57	58	105.17	93.41	138.55
58	s/n	116.32	101.57	128.39	58	59	–	–	–
59	s/n				59	60	101.09	–	–
60	1			127.65	60	61	103.22	–	–
61	2		103.57		61	62	103.69	95.44	139.37
						63	99.38	94.36	142.96
						64	96.3	103.2	157.6
**KUVP 1301 (*Styxosaurus snowii*)**					**AMNH 2554 (‘*Cimoliasaurus magnus*’)**				
1	3	23	25	–	1		68,64	61,26	88,14
2	4	30	27	–	2		69,62	66,48	89,95
3	6	48	42	–	3		70,33	64,11	88,93
4	9	53	44	–	4		70,16	63,66	88,63
5	14	63	50	–	5		70,82	67,87	91,34
6	20	78	60	–	6		72,67	72,92	95,04
	27	90	68		7		72,34	76,78	100,43
					8		74,67	84,86	109,19
					9		75,55	84,04	111,84
					10		73,37	–	119,91

**Table:** 

*STYXOSAURUS* SP.

Figs. 2–6

**Table:** 

*Elasmosaurus serpentinus*: In Cope, 1877; Williston, 1903; Williston, 1906.
*Elasmosaurus sergentinus*: In Watson, 1924.
*Hydralmosaurus serpentinus*: In Welles, 1943; Welles, 1949; Welles, 1952;Welles, 1962; Welles & Bump, 1949; Persson, 1960; Persson, 1963; Kuhn, 1964; Carpenter, 1999.

**Referred specimen**—AMNH 1495. An almost complete axial skeleton lacking two or three centra, partial pectoral and pelvic girdles, and both hindlimbs.

**Locality, horizon and age**—Following Cope (1877), collected from blue shales on the southwest side of the Missouri River, between Sioux City, Iowa, and Yankton, Dakota. Lower Pierre Shale Group, Sharon Springs Formation, *Baculites obtusus*—*Baculites perplexus* biozone, middle Campanian (Welles, 1943; Martin, Bertog & Parris, 2007; Bertog, 2010).

**Taxonomical determination**—overlapping material between AMNH 1495 and KUVP 1301 (holotype of *S. snowii*) only includes their cervical vertebrae. On the other hand, the completeness of AMNH 1495 permits a good comparison with *S. browni* (AMNH 5835; holotype, below), as both have several relevant differences that support them as different taxa. AMNH 1495 differs from *S. browni* (AMNH 5835) in the following features: a slightly smaller adult skeleton, a glenoid process of the scapula less expanded than that of *S. browni*, a scapular shaft shorter than that of *S. browni*, and transverse processes of dorsal vertebrae that are laterodorsally oriented on AMNH 1495 while in *S. browni* these are almost horizontal.

**Table:** 

*STYXOSAURUS BROWNI* Welles, 1943

Figs. 7–11

**Table:** 

*Styxosaurus browni*: In Welles, 1952; Welles, 1962; Persson, 1963; Kuhn, 1964.
*Hydralmosaurus serpentinus*: In Carpenter, 1999


**Holotype**—AMNH 5385. Skull, complete neck, anterior trunk and left forelimb.

**Locality, horizon and age**—Mule Creek, 15 miles west of Edgemont, South Dakota, USA. Lower Pierre Shale Group, Sharon Springs Formation, *Baculites obtusus*—*Baculites perplexus* biozone, middle Campanian (Welles, 1943; Martin, Bertog & Parris, 2007; Bertog, 2010).

**Differential Diagnosis**—AMNH 5835 has a jugal bar comparatively higher in its posterior margin, with a well-marked temporal ridge on its medial aspect, and its anterior part is dorsoventrally narrower just behind the orbit, as opposed to *S. snowii* (KUVP 1301), whose jugal bar is more squared and the temporal ridge is shallower. Preorbital boss is rounder and larger than that of *S. snowii*; dorsal contact of squamosals is not prominent with respect to the sagittal crest, as it occurs on *S. snowii*; height of the posterior process of the maxilla reaches the half of the jugal bar, while in *S. snowii* this is one third the height of the jugal bar.

## Osteological Description of AMNH 1495, *STYXOSAURUS* SP.

**Ontogenetic stage**—AMNH 1495 is referred to an adult based on the lost neurocentral sutures in the axial skeleton, as well as on the well-defined facets of the humerus, epipodials and distal carpals. Such features have been considered as indicative of an adult stage (Brown, 1981).

**Figure 2:**
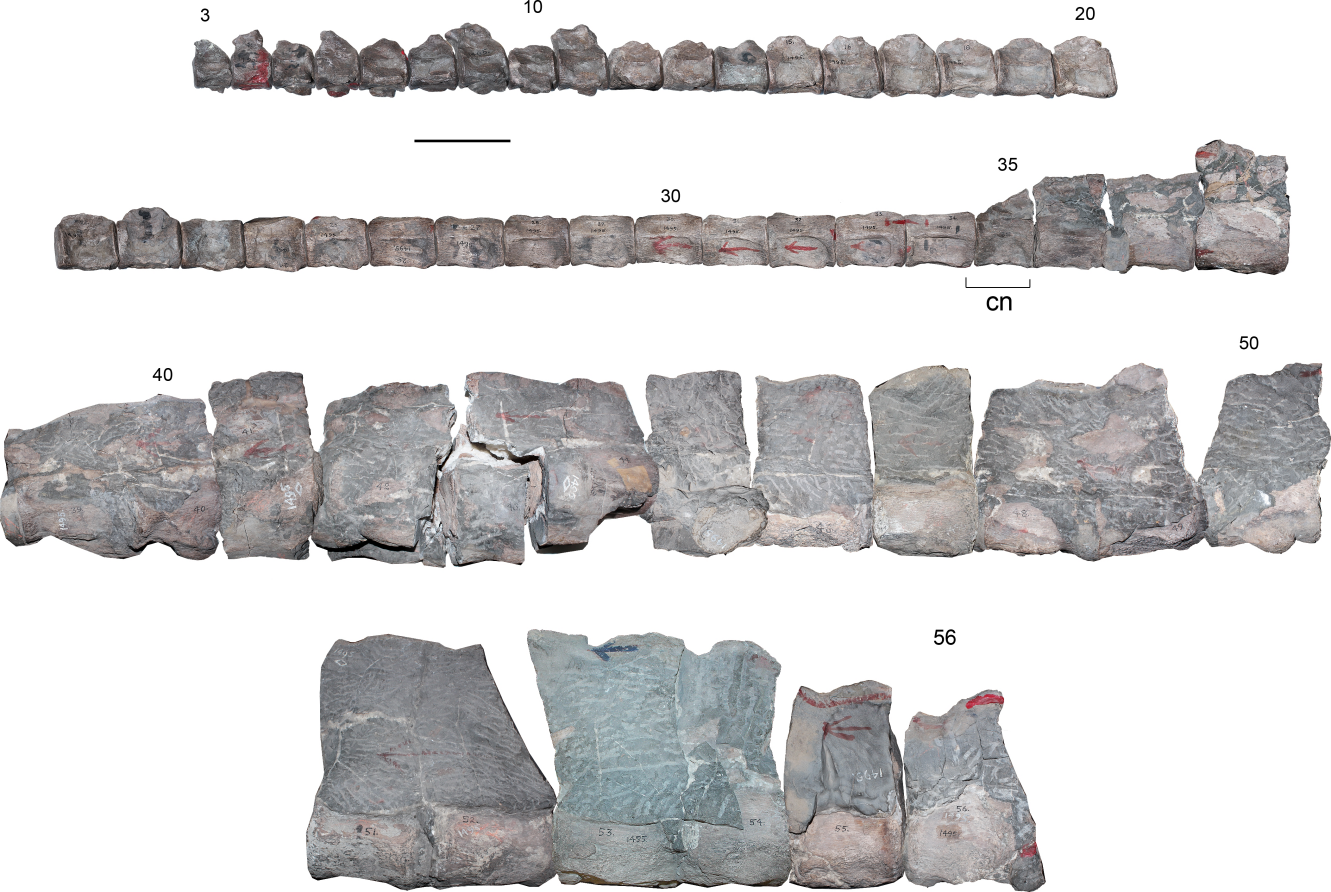
*Styxosaurus* sp. (AMNH 1495). Cervical vertebrae in left lateral view. Numeration follows the original designation of Welles (1943). Cervicals 39–56 are partially covered by matrix. Anatomical abbreviations: cn, cervical node. Scale bar equals 10 cm.

**Figure 3:**
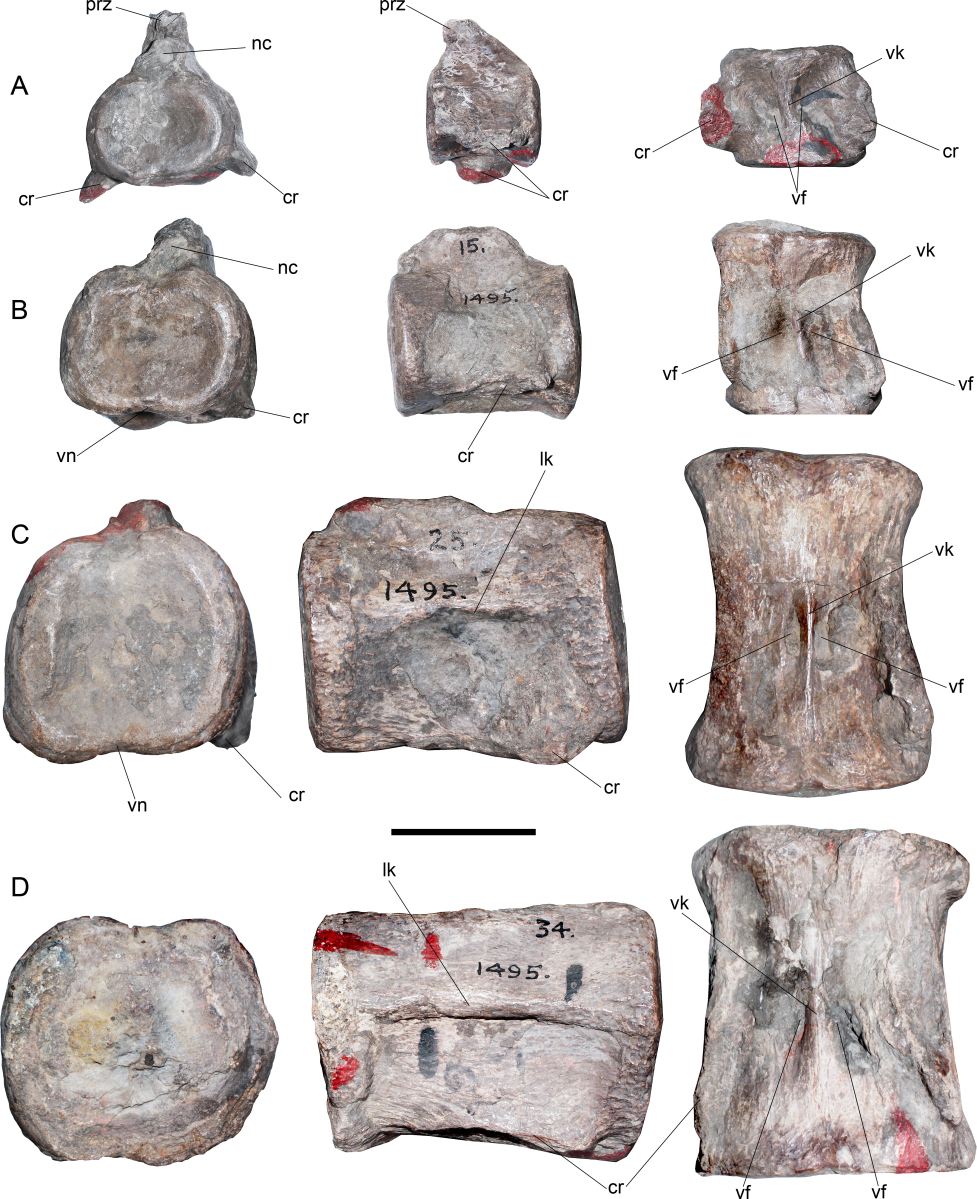
*Styxosaurus* sp. (AMNH 1495). Detail of cervicals. (A) c6 on anterior, left and ventral views, respectively. (B) cervical 15 on the same views. (C) cervical 25 on the same views. (D) cervical 34 on the same views. Numeration follows the original designation of Welles (1943). Anatomical abbreviations: cr, cervical ribs; nc, neural canal; lk, lateral keel; prz, prezygapophysis; vf, ventral foramina; vk, ventral keel. Scale bar equals 5 cm.

**Cervical vertebrae**—AMNH 1495 anterior cervical vertebrae do not preserve any complete cervical rib, while neural arches are mostly incomplete (Fig. 2). The latter are much narrower than the centrum and they have a neural canal that is dorsoventrally short and circular. A remarkable feature on the neck is observed in c35, which marks a morphological inflection between the anterior cervical centra and the rest of the neck (Fig. 2). Between c3 and c10 the proportions of each centra are similar. Around c6 and c7, a soft lateral keel appears. These centra are broader than high and as high as long, with a ventral notch that gives them a bilobed articular outline. On ventral view, the two foramina are short and separated by a ventral keel. The articular facets are slightly prominent with respect to the body of the centrum, having axial striations surrounding the articular facet on their lateral and ventral sides. Centra are mostly platycelous, while anteriormost elements are slightly amphicelous (Figs. 3A and 3B). From c11 to posterior, a progressive increase of the centrum length is noted. Around c24–c34, the length of each centrum reaches near twice their respective height (or width). Also, lateral keels of each centrum become well-marked (Figs. 3C and 3D).

These centra are remarkably unique among elasmosaurids. Outside the Western Interior Seaway such cervical proportions remain unreported (see ‘Discussion’). A cervical node is visible on c35 (Fig. 2). This centrum is remarkably shorter than the previous ten anterior centra. A marked change is also present on the lateral keel, which becomes shallower. From c36 backwards, the length of each centrum again increases, being one third larger than c35 length. Also, the preserved neural spines show a blade-like lateral outline are placed over each centra without any anterior or posterior shifting (as it occurs on anterior cervical vertebrae of AMNH 5835). From c39 onwards, cervical vertebrae are mostly covered by sediment, still, they show elongated centra and progressively higher neural spines that reach twice the centrum height on their posterior elements. All the centra with their ventral portion visible exhibit a ventral keel that separates the ventral foramina. The last numbered cervical vertebra is c56, followed by five vertebrae preserved in two blocks and obscured by the sediment. Thus, the final cervical numeration should be c61, but, taking into consideration that the cervical count of AMNH 1495 starts at c3, 58 cervical vertebrae are actually known for this specimen.

**Pectoral vertebrae**—Pectoral vertebrae (*sensu* Sachs, Kear & Everhart, 2013) were not found among the available material.

**Figure 4:**
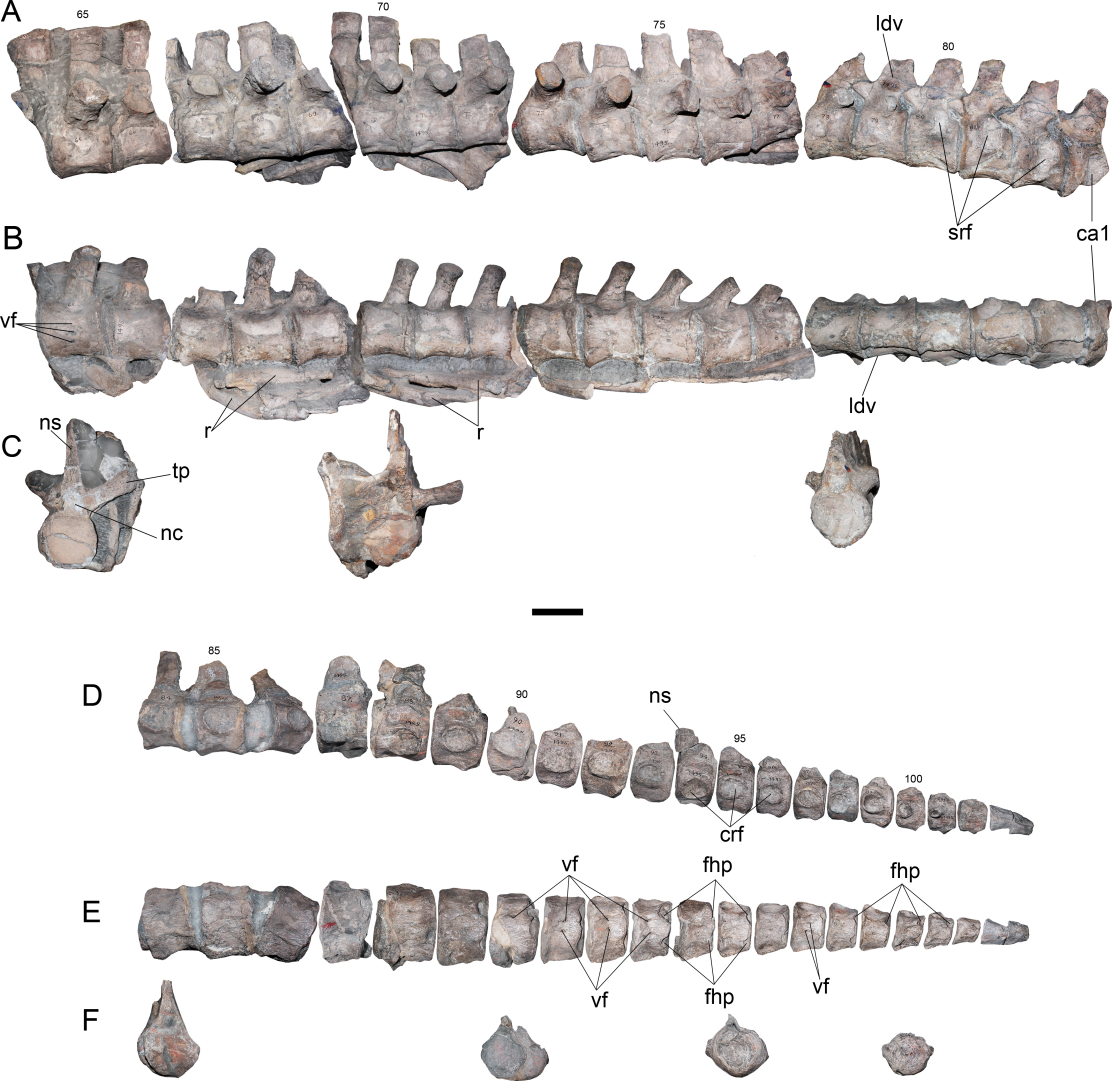
*Styxosaurus* sp. (AMNH 1495). Dorsal and caudal series of the axial skeleton. Dorsal series. (A) left lateral view. (B) ventral view. (C) From left to right, posterior view of dorsal 87, anterior view of dorsal 70 and anterior view of dorsal 78. Caudal series. (D) left lateral view. (E) ventral view. (F) anterior views of caudal 84, 90, 95 and 100. Numeration follows the original designation of Welles (1943). Anatomical abbreviations: crf, caudal rib facets; fhp, facet for the haemal processes; ns, neural spine; ldv, last dorsal vertebra; nc, neural canal; r, ribs; srf, sacral rib facets; tp, transverse process; vf, ventral foramina. Scale bar equals 10 cm.

**Dorsal vertebrae**—15 dorsal vertebrae are preserved on five different blocks. The anteriormost dorsal centrum is numbered 65, implying that only three vertebrae (62–64) are missing. Considering the height of the dorsal processes on vertebra 65, which are far from the neurocentral suture (Fig. 4A), plus the three absent vertebrae 62–64, it is evident that the current numeration is likely omitting two or three centra, which are pectoral or anterior dorsal verterbrae. Considering these absent elements, the dorsal count reaches 17 or 18 centra. Morphologically, dorsal vertebrae of AMNH 1495 are typical of elasmosaurids, with centra as long as broad as high, neural arches narrower than the centrum, robust transverse processes, short and suboval neural canali and high neural spines (Fig. 4A and 4B). The dorsal vertebra 65 shows transverse processes with 30°–35°of inclination with respect to the horizontal axis (Fig. 4C). Also, the neural spines have near 1.5 times the height of the centrum, with an anterior triangular cross section that indicates a blade-like dorsal edge instead a flat top. Articular facets are rounded. From the dorsal vertebra 70 and posteriorly, the transverse processes are comparatively less robust than those of the anterior dorsal vertebrae. These also have a lower angle, close to 20°. In addition, the transverse processes are progressively recurved backwards on posterior dorsal vertebrae, while neural spines become shorter and thicker.

**Figure 5:**
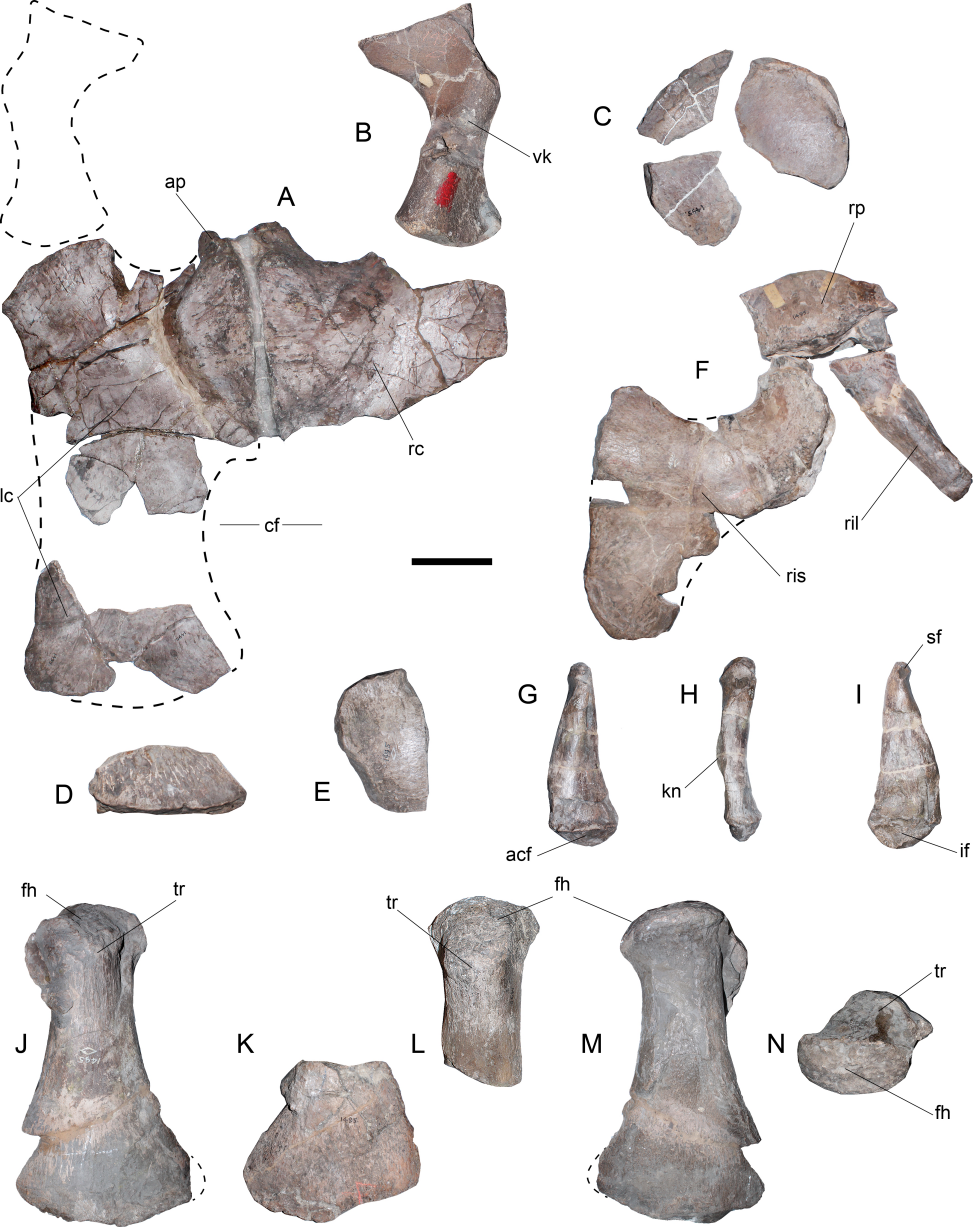
*Styxosaurus* sp. (AMNH 1495). Pectoral and pelvic girdle elements; femora. (A) coracoids in dorsal (internal) view. (B) left scapula in ventral view (only available view). (C–E) fragments of pubes. (F) right acetabular portion with the near complete ischium, the right ilium and the acetabular fragment of the right pubis. (G) right ilium in external (right lateral) view. (H) same on anterior view. (I) same on internal view. (J) dorsal view of the left femur. (K) dorsal view of right femur for comparison. (L) dorsal view of the proximal part of the right femur. (M) ventral view of the left femur. (N) proximal view of the left femur, partially crushed. Anatomical abbreviations: acf, acetabular facet; ap, anterior process; cf, cordiform fenestra; fh, femoral head; if, ischiadic facet; kn, knee; lc, left coracoid; rc, right coracoid; ril, right ilium; ris, right ischium; rp, right pubis; sf, sacral facet; tr, trochanter; vk, ventral keel. Scale bar equals 10 cm.

**Sacral vertebrae**—Three articulated sacrals are preserved, numbered 80–82. These centra are longer than broad and as broad as high, with rounded articular facets. The rib facet passes from a ventral short rib facet, dorsoventrally higher than axially long, to an ‘eight shaped’ facet that occupies nearly two thirds of the lateral surface of the centrum. On these vertebrae, the neural spines are short and have a squared outline.

**Caudal vertebrae**—The first caudal is attached to the same block as the sacrals, being numbered as 83. The rest of the caudal vertebrae are preserved in a block including elements 84–86, while the remaining centra are separated from the matrix. A total of twenty-two caudal vertebrae are here recognized. The anterior caudal vertebrae are as broad as high and as long as broad, with a slightly hexagonal outline, a rounded and reduced neural canal, and short neural spines with an anterior triangular cross-section. On lateral view (Fig. 4D), the caudal rib facets become progressively ventralized until centrum 88, where these appear in a more dorsal position. From centrum 88 backwards, these facets descend again and migrate anteriorly from centrum 98–101, where finally they fade. Ventrally, from centrum 86 and backwards, there is a marked pair of facets towards the haemal processes. These facets are placed on the ventroposterior articular margin of each centrum. From centrum 91 backwards, a pair of shallow anterior facets for the haemal processes appear on each centrum, showing that haemal processes were placed between each centrum (Fig. 4E). The caudal articular facets are progressively depressed in the dorsoventral direction, while in the last caudal centra they acquire a nearly squared outline (Fig. 4F). The last two caudal vertebrae, c103 and c104, are attached together.

**Coracoids**—Although the pectoral girdle is fragmentary, the coracoids can be interpreted based on the available portions (Fig. 5A). A good part of the anterior portion of both coracoids retains its anatomical position. The left coracoid is the most complete and shows a straight midline where it joins the right coracoid. The anteromedial process is laterally curved and does not extend far beyond the glenoid, thus, not having any medial contact with the scapula (pectoral bar absent). A ventral process is present in both coracoids adjacent to the midline. The glenoid facet is poorly differentiated from the scapular facet. Both facets are anteriorly oriented in ca. 45°with respect to the axial direction. Medially, a small portion of the coracoid outline is complete, showing the lack of medial contact with other elements. The posterior part of the left coracoid is identified based on its ventral concavity and dorsal convexity. This preserves part of the posterior outline. These portions contribute to verifying the presence of an open cordiform fenestra between the posterior end of both coracoids.

**Scapula**—Only the posterior portion of the left scapula is preserved (Fig. 5B) and still embedded in the matrix. This is available only in a ventral view, showing the presence of a ventral keel, a gracile shaft and a posterior margin expanded about two thirds of the shaft breadth. The articular facets for the coracoid and for the glenoid are poorly differentiated. Together with the coracoid, both elements form a glenoid narrower than the articular head of the humerus.

**Pubis**—The pubes are very fragmentary and their preserved portions do not allow understanding their outline. Three fragments suggest a rounded anterolateral margin (Fig. 5C), however, this cannot be assured. Additional remains (Fig. 5D) belong to the ischial facet and likely, to a lateral cornua. The posteriormost portion of the right pubis can be attached to the right ischium and ilium.

**Ischium**—The acetabular fragment of the left ischium (Fig. 5E) and most of the right ischium are preserved (Fig. 5F). The ischium is as long as broad, with a shallow transverse process extended between the midline and the acetabulum. The articular facet for the pubis is recurved with respect to the transverse process. The posterior end of the ischium is flattened and has a rounded contour.

**Ilium**—The right ilium is the only one preserved (Figs. 5G–5I). This element is remarkably distinctive from other elasmosaurids due to the presence of a triangular outline from an internal view, with a expanded ventral margin and a very narrow dorsal end (Fig. 5G). It can be seen that the shaft is slightly sigmoidal, while the dorsal articulation for the sacral ribs is recurved. The ventral end has a large articulation for the ischium. A medial knee is visible over the external surface on an anterior view (Fig. 5H). Both the sacral and the pubic facets are visible from an internal view (Fig. 5I).

**Hindlimbs**—Although the left femur (Figs. 5J, 5M and 5N) is dorsoventrally crushed, it is possible to asses that it matches the outline and general shape of the right femur. Proximally, the trochanter is identical in shape, and in both cases it is slightly shifted anteriorly with respect to the axial midline. The distal facets are identical, considering that a small fragment of the fibular facet is missing. The articular head of the left femur is evidently crushed (Fig. 5N). This condition affected most of the shaft. The crushed shaft was re-attached to the undeformed distal end, causing a prominent edge on the preaxial margin of the shaft, which is an artifact caused by the different degree of compression on each part of the bone.

**Figure 6:**
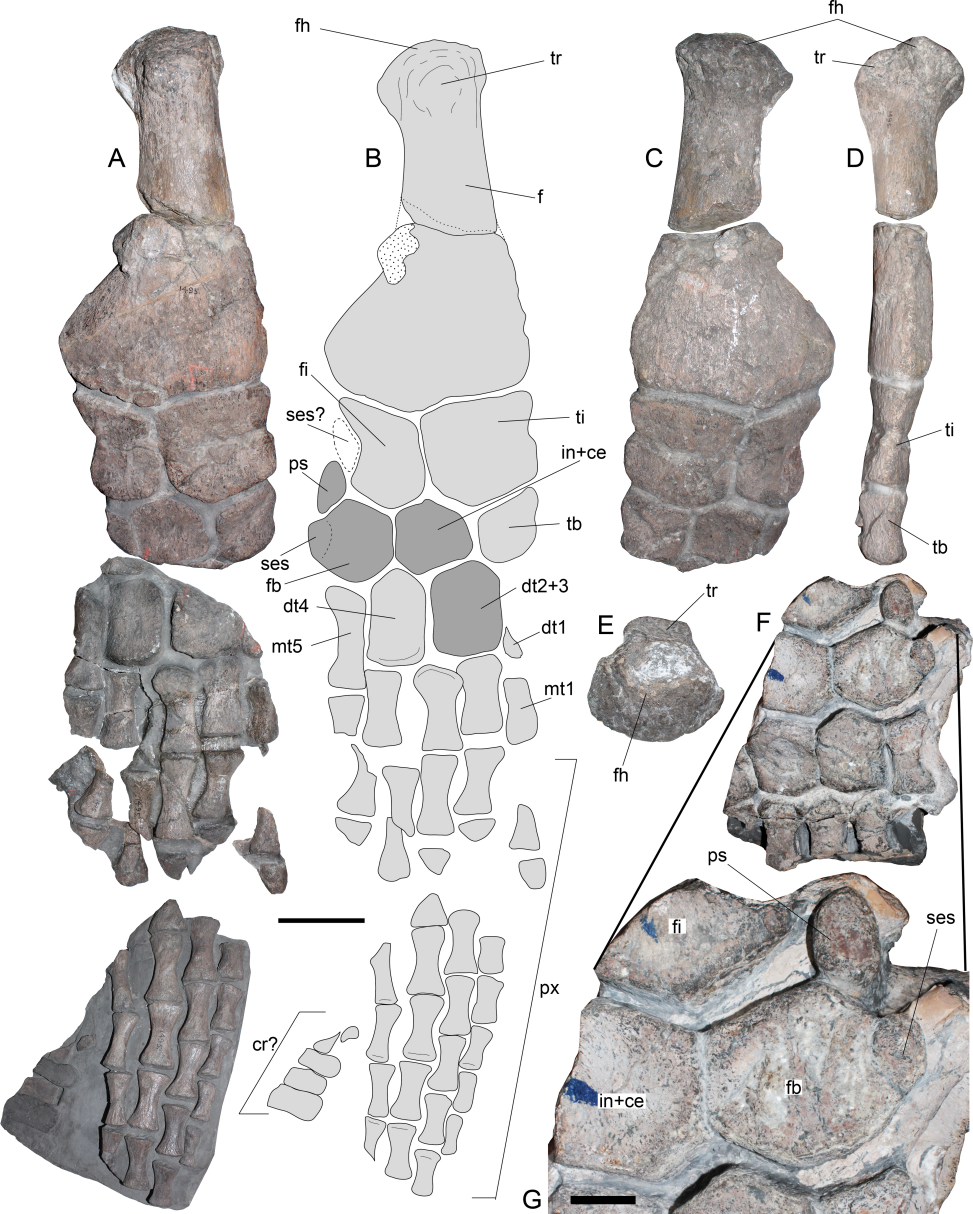
*Styxosaurus* sp. (AMNH 1495). Hindlimbs. (A) Left hindlimb in dorsal view. (B) interpretation of the same. Dark grey elements are interpreted based on the left hindlimb. (C) ventral view of the block hosting the left femur, their epipodials and mesopodials. (D) same on posterior view. (E) proximal view of the articular head of the left femur. (F) preserved part of the right hindlimb. (G) close-up of the ulnare fused to a sesamoid. Anatomical abbreviations: cr?, caudal ribs?; dt1, distal carpal 1; dt2 + 3, distal carpal 2 + 3; dt4; distal carpal 4; f, femur; fh, femoral head; fi, fibula; fb, fibulare; mt1, metatarsal 1; mt5, metatarsal 5; ps, pisiform; px, phalanges; ses, sesamoid; ses?, expected sesamoid; ti, tibia; tb, tibiale; tr, tuberosity. Scale bar equals 100 mm; except (G), 10 mm.

Most of the right hindlimb is preserved in three major blocks (Fig. 6A and 6B). The right femur remains articulated with the epipodials and the distal tarsal elements, while additional proximal and distal elements are in separate blocks. The right femur (Figs. 6A–6E) has a straight shaft and expanded distal facets. The latter are slightly concave for articulation with the respective epipodials. Proximally, the femur has an articular head prominent in comparison to the shaft and a dorsally prominent trochanter (Figs. 6C–6D). The tibia is slightly longer than broad, with a notched preaxial margin, while the postaxial margin is straight. The fibula has remarkable features. It is longer than broad, with a straight preaxial margin and a deeply concave postaxial margin. An epipodial foramen is lacking between these two elements. The tibiale, part of the central element (here interpreted as the intermedium + centrale) and part of the fibulare are preserved in the same block. Nonetheless, the latter two are better preserved in the available portion of the left hindlimb (Fig. 6F). The tibiale preserved on the femur block shows a rhomboidal shape and it is as long as it is broad. The intermedium + centrale on the left hindlimb has at least four facets between the fibula and the tibia. The outline of this element is polygonal, as broad as it is long. The fibulare is fused to a sesamoid in its postaxial margin (Fig. 6G). In addition, a pisiform is settled over the postaxial margin between the fibula and fibulare. This pisiform has a circular anterior outline and a straight posterior margin. Prior to the fusion of the sesamoid, the fibulare likely had a concave postaxial margin, as it occurs with the fibula. The preserved pisiform is articulated and placed in the postaxial margin between the fibula and fibulare, leaving an unusual gap on the postaxial margin of the fibula. Furthermore, if this element actually belonged to a sesamoid articulating with the fibula, the postaxial gap between fibula and fibulare would be even larger. Based on the good preservation and articulation of the left hindlimb portion, this research considers that the pisiform is indeed articulated and not displaced, while a missing element, likely a sesamoid, is absent on the postaxial margin of the fibula. Distal tarsals 2 + 3 and 4 are well preserved. These are axially longer than broad, having both a squared outline and a pair of proximal facets for articulation with the fibulare, intermediate + centrale and tibiale. Finally, the phalanges are spool-shaped, elongated, and dorsoventrally flattened.

**Figure 7:**
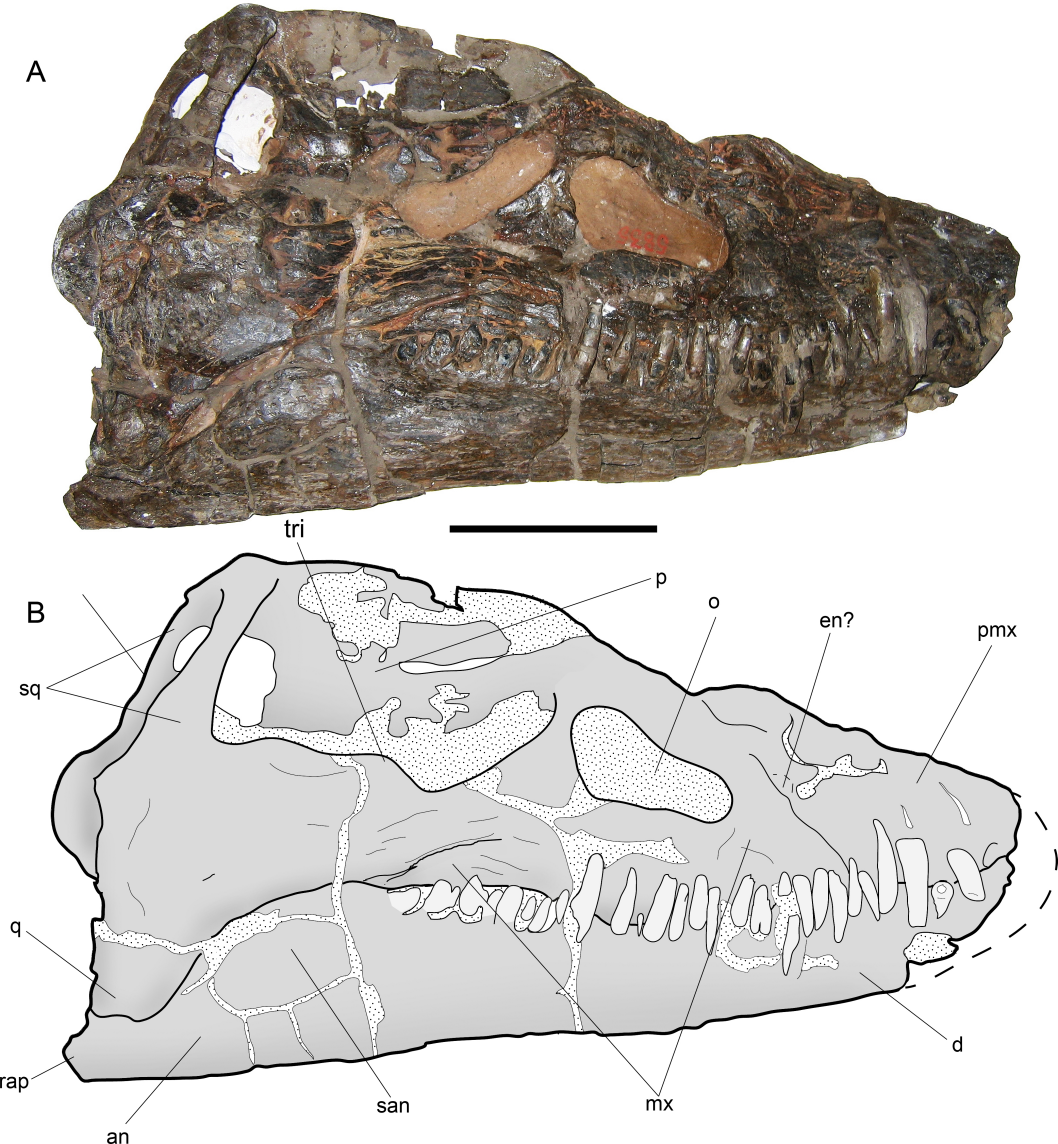
*Styxosaurus browni* (Welles, 1952) (AMNH 5835). Skull. (A) right lateral view. (B) interpretation of the same. Anatomical abbreviations: an, angular; d, dentario; en?, external naris?; mx, maxillar; o, orbit; p, parietal; pmx, premaxillar; q, quadrate; rap, retroarticular process; san, surangular; sq, squamosal; tri, temporal ridge. Scale bar equals 10 cm.

## Osteological Description of AMNH 5835, *STYXOSAURUS BROWNI* Welles, 1943

**Ontogenetic stage**—The skull sutures are mostly lost. In addition, the humeri and epipodials have well-defined facets. The axial skeleton has neurocentral sutures lost. All these characters suggest an adult stage for AMNH 5835, following the criteria of Brown (1981).

**Skull**—The skull of AMNH 5835 (Figs. 7A and 7B) is laterally crushed and only visible on right view. The anteriormost part of the rostrum and dentaries are lost. General features are the presence of a large temporal fossa having near one third of the skull length, and the orbit settled in the middle part of the skull. The orbit has a reniform outline and its ventral margin is convex. The temporal fossa has a squared contour with a medial ridge over its lateral margin. The anterior part of the fossa extends just posterior to the orbit, separated by a bony bridge, likely the postorbital. Most sutures are difficult to see. The most evident suture is the contact between the maxillary and the rest of the jugal bar. From the posterior margin of the orbit, the posterior extension of the maxilla is equivalent to the orbit length. The jugal bar has its narrower part anteriorly, becoming thicker towards its posterior end. The squamosal arch is completely preserved. Each squamosal dorsal process is axially compressed. The squamosals meet at the midline and form a dorsal boss on the dorsal part of the skull. The posterior part of the skull has no visible sutures. The posterior margin of the left squamosal is partially visible, showing a posteriorly projected medial boss. This part is broken in the right squamosal. The quadrate cannot be delimited because it is strongly fused to the squamosal. The sagittal crest is damaged, although the preserved portions show that it was moderately high and dorsally projected to the orbital roof. Anterior to the orbit, a partial suture marks the contact between the maxillary and premaxillary. Near this suture the bone is cracked and few parts are missing, encumbering the recognition of the external naris, however, no other anatomical cavity is evident in this portion, indicating that the external naris is likely anterior to the maxillary-premaxillary suture. The maxillary preserves thirteen teeth. Tooth preservation is variable, with a few of them still on anatomical position and keeping their crowns, while others are broken, eroded or missing. Few teeth show part of the lingual enamel, and in most cases, this is eroded. The lingual enamel has thin, soft and profuse striations. The longest preserved teeth occur anterior to the maxillary-premaxillary suture, and below the posterior margin of the orbit. The occlusal margin of the dentary is slightly sigmoidal, with a high coronoid process. Posterior to the coronoid process, the mandibular ramus is lower than the tooth row. Even though the posterior part of the mandibular ramus is cracked, at least two cracks are coincident with the sutures between surangular-angular, and between these and the dentary. The retroarticular process is incomplete.

**Figure 8:**
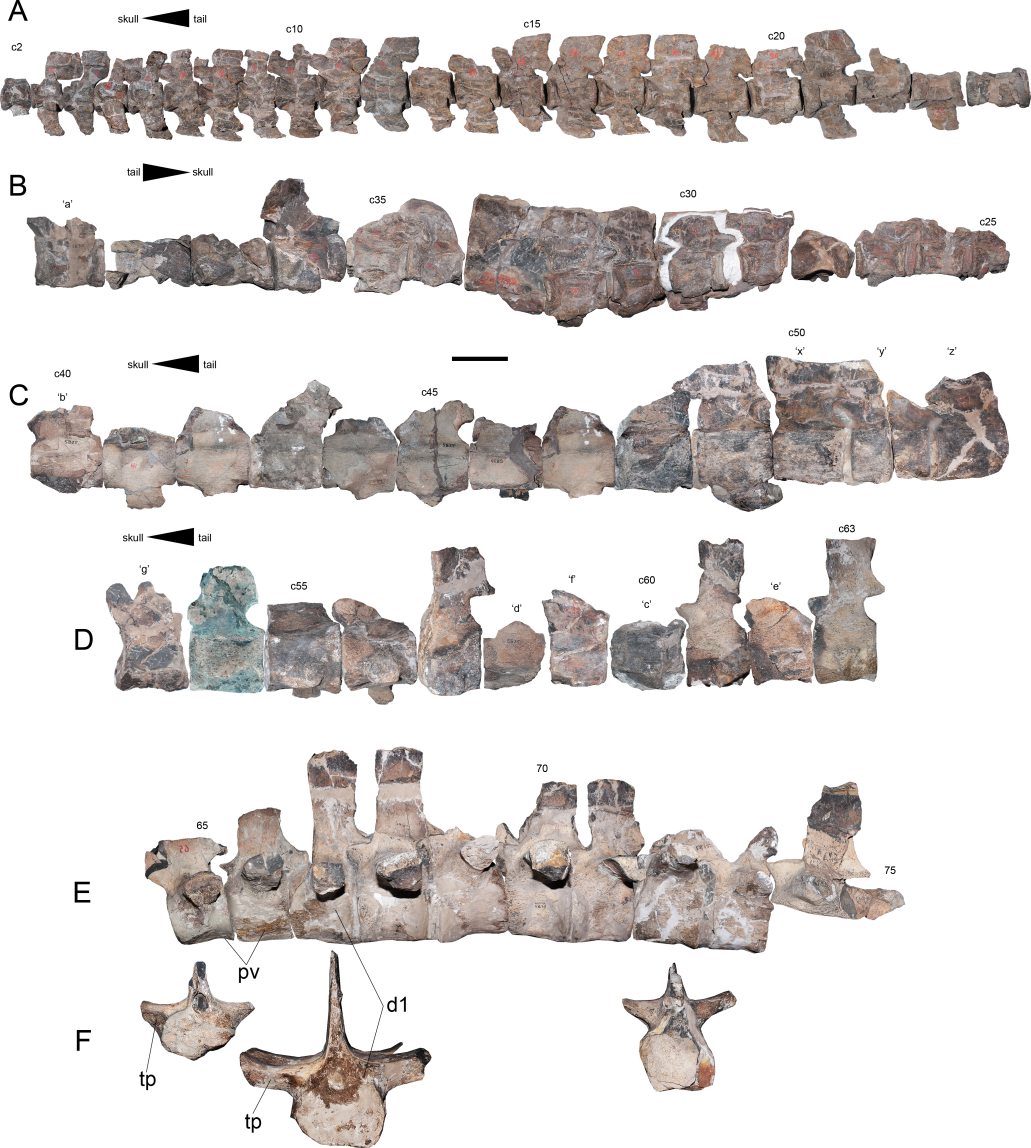
*Styxosaurus browni* (Welles, 1952) (AMNH 5835). Cervical, pectoral and dorsal series of the axial skeleton. (A) First 23 articulated cervicals (c2–c24) on left lateral view. (B) 15 following cervicals (c25–c39) on right lateral view (best preserved view for this portion). (C) cervicals c40–c52 on left lateral view. (D) cervicals c53–c63 on left lateral view. (E) Last pectorals and dorsal vertebrae on left lateral view. (F) pectoral vertebra 65, dorsal vertebrae 67 and 72, all on anterior view. Dorsal 65 is mirrored for better view. Numeration follows the original nomenclature of Welles (1943). Unnumbered centra or centra with uncertain position are labeled with letters and reordered considering their measurements (Table 4). Anatomical abbreviations: d1, first preserved dorsal; pv, pectoral vertebrae; tp, transverse process. Scale bar equals 10 cm.

**Cervical vertebrae**—The cervical vertebrae are numbered from c2 to c63 (Figs. 8A–8D). Most cervical vertebrae are free from the matrix, while a few centra remain embedded and hosted in several blocks. Due to this, c29–c35 are better observed on right view. The anterior cervical vertebrae are well-preserved, keeping their ribs and neural arches. These centra are longer than broad and as high as broad. The cervical ribs are blade-like, recurved posteriorly and they have shallow anterior processes and more extended posterior processes. In at least in the first 21 centra, the neural spines are short and have a squared outline from a lateral view. On anteriormost cervical vertebrae, the neural spine overhangs each posterior centrum by a remarkable extension of the postzygapophyses, while the prezygapophyses are short. From c10 to c15, the prezygapophyses become larger, while the posterior projection of each neural spine becomes shorter. From c20 and backwards, the neural spines are almost completely equivalent in length to the centrum, while their height is about one third larger than the centrum height. From c16 to c47, there is a drastic increase in the length with respect to the anteriormost cervicals. Several cervical vertebrae of this section are ‘can-shaped’, being similar to those described on AMNH 1495. Vertebra c47 is shorter than any surrounding element, suggesting the presence of a cervical node (Fig. 8C). Posterior to c48, the centra become progressively broader than long and they are all longer than high. Ten unnumbered centra were relocated based on their measurements. Few of them are fragmentary and likely belong to the posteriormost cervical vertebrae or else, to the pectorals (Fig. 8D). A total of 63 pre-dorsal vertebrae are identified in this review.

**Pectoral vertebrae**—Two unambiguous pectoral vertebrae were recognized based on the articulation of the rib in an intermediate position between the centrum and the neural arch. These pectoral vertebrae are numbered 65 and 66, respectively (Fig. 8E). The presence of at least one additional pectoral among unidentified pre-dorsal centra can be expected.

**Dorsal vertebrae**—Seven dorsal vertebrae and fragments of two additional dorsal vertebrae are identified. Among the best preserved elements it is possible to distinguish the presence of robust transverse processes which are oriented almost horizontal (Fig. 8F), condition which is retained at least until vertebra 71. From dorsal vertebra 72 backwards, the transverse processes are more gracile and they have a slight dorsal recurving. The ventral surface of all dorsal elements is damaged, being impossible to evaluate them. However, besides the direction of the transverse processes, the dorsal vertebrae of AMNH 5835 do not show additional distinctive features from other elasmosaurids.

**Figure 9:**
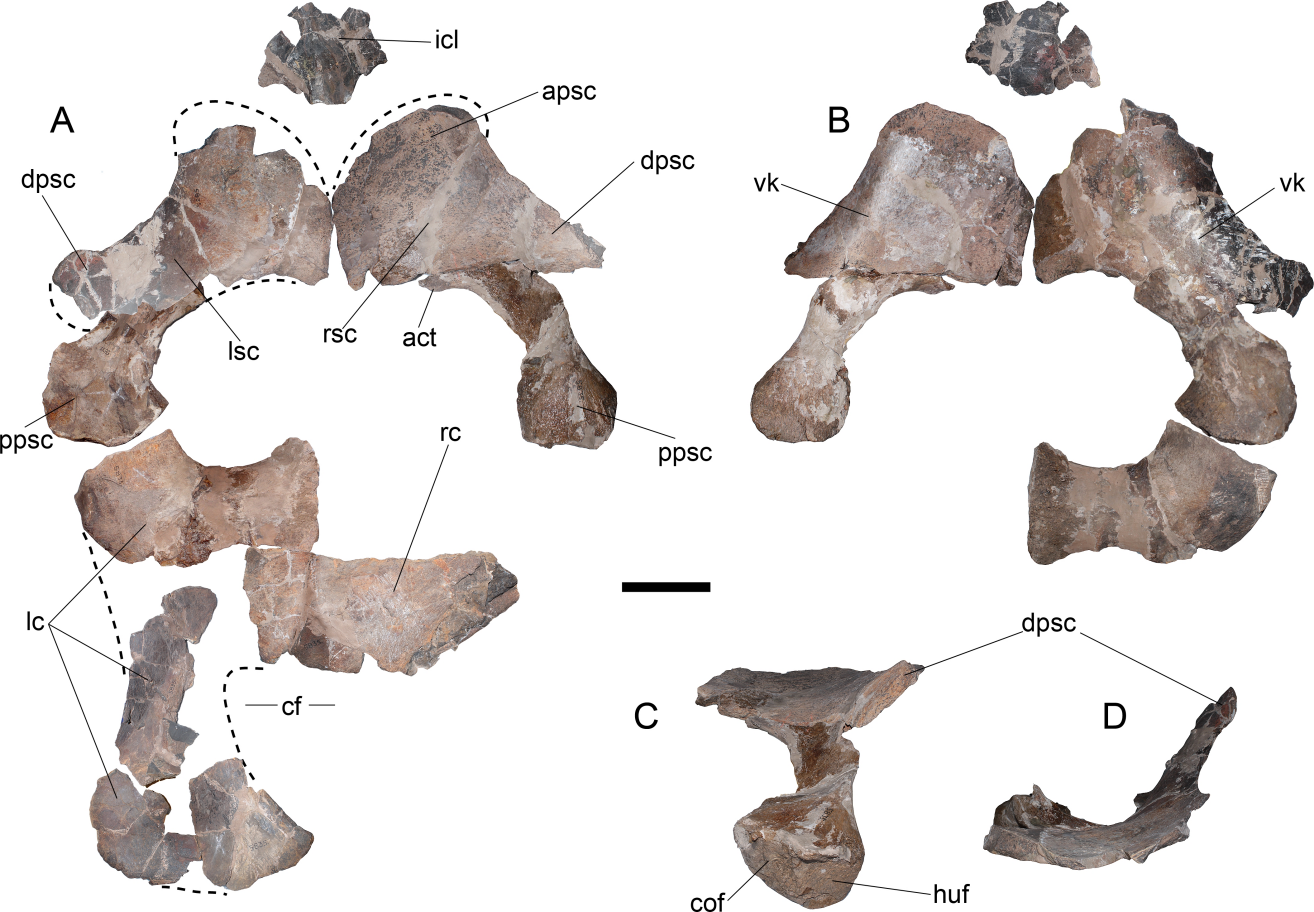
*Styxosaurus browni* (Welles, 1952) (AMNH 5835). Pectoral girdle. (A) dorsal view. (B) ventral view. (C) dorsoposterior view. (D) anterior view. Anatomical abbreviations: act, acromion tuberosity; apsc, anterior process of the scapula; cf, cordiform fenestra; cof, coracoidal facet; dpsc, dorsal process of the scapula; huf, humeral facet; icl, interclavicular; lc, left coracoid; lsc, left scapula; ppsc, posterior process of the scapula; rsc, right scapula; vk, ventral keel. Scale bar equals 10 cm.

**Pectoral girdle**—The coracoids are represented by an anterior portion and by few posterior fragments of the left one, and part of the symphyseal contact between both (Fig. 9A). Two portions allow observing most of the midline contact. There is a small anterior process that lacks contact with the scapula at the midline (no pectoral bar). It is difficult to evaluate the presence of a ventral process since the coracoid midline is dorsoventrally crushed. The posterior outline of the left coracoid is partially preserved, showing the presence of a cordiform fenestra. The posterolateral margin is rounded while the internal margin, which forms the cordiform fenestra, has a prominence. Anteriorly, the contact with the scapula leaves a narrow glenoid. The left scapula is preserved on a few fragments (Fig. 9A). It has a posterior process conformed by a recurved shaft with a shallow ventral keel (Fig. 9B). Its posterior end is expanded around twice the shaft’s breadth, having two well-marked facets, one towards the coracoid and the other towards the glenoid (Fig. 9C). The dorsal process is blade-like and shorter than the ventral process (Fig. 9D).

**Figure 10:**
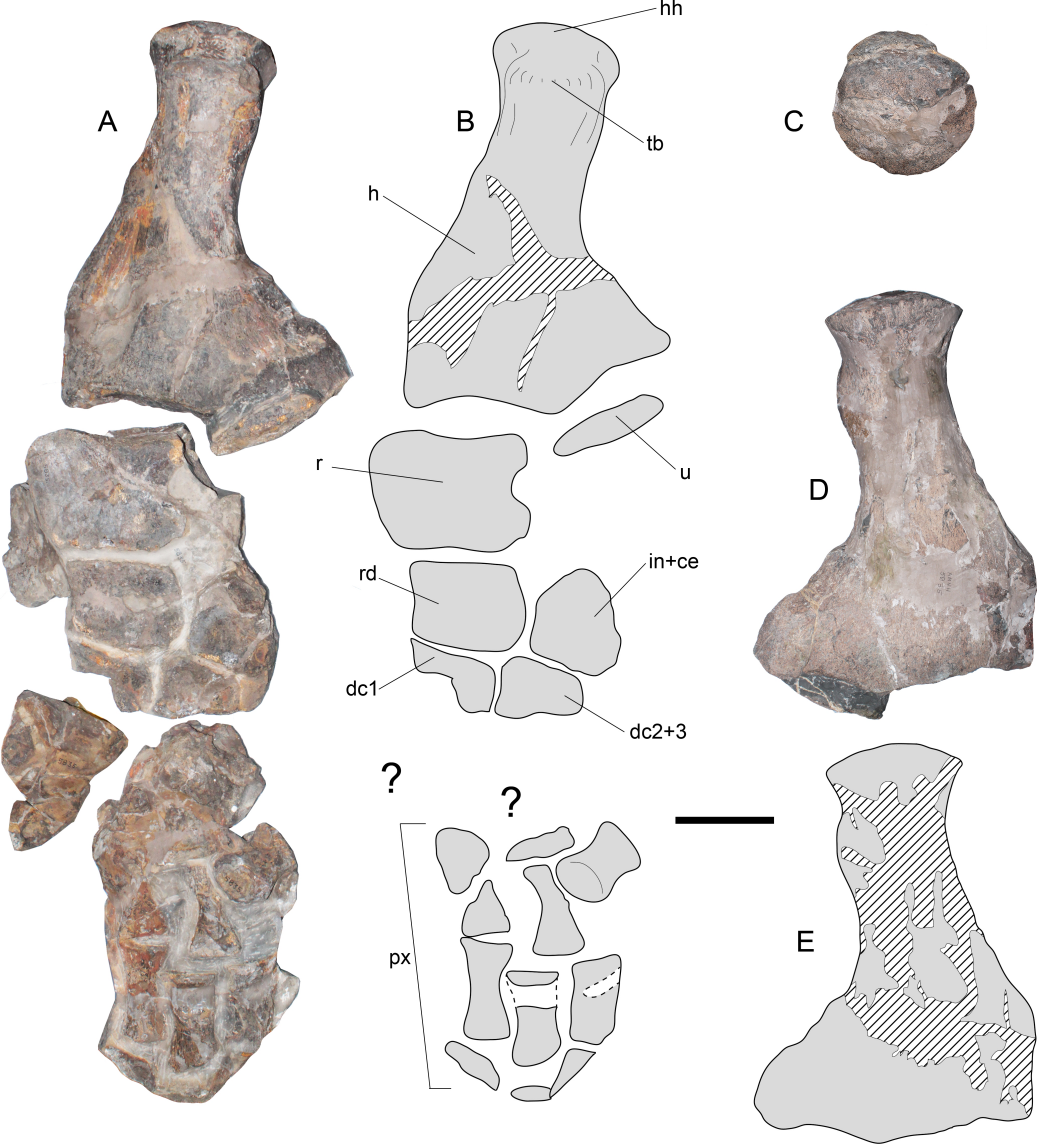
*Styxosaurus browni* (Welles, 1952) (AMNH 5835). Left forelimb (A) dorsal view. (B) interpretation of the same. (C) humeral head in proximal articular view. (D) left humerus in ventral view. (E) interpretation of the same. Anatomical abbreviations: hh, humeral head; dc1, distal carpal 1; dc2 + 3, distal carpal 2 + 3; h, humerus; in + ce, intermedium fused with centrale; px, phalanges; r, radius; rd, radiale; tb, tuberosity; u, ulna. Scale bar equals 10 cm.

**Forelimb**—The left forelimb lacks its distal phalanges (Figs. 10A and 10B). The humerus shows a sigmoidal shaft, however, a good part of the shaft has been reconstructed, making it difficult to assure whether this feature is real or an artifact of the reconstruction. From a proximal view (Fig. 10C), the humeral head is rounded, while the tuberosity is prominent with respect to the former. Distally, the humerus has two well marked, concave articular facets. On dorsal view, both the radial and ulnar facets appear similar in length, however, from a ventral view (Figs. 10D and 10E) the radial facet appears larger. This could be the effect of taphonomic distorsion. The radius is as long as broad, with a convex preaxial margin and a medial notch on its postaxial margin. The ulna is represented by a small proximal fragment insufficient for the evaluation of its outline. The radiale has a subrectangular outline with its preaxial margin slightly concave. The anterior half of the intermedium + centrale is also preserved, showing distinctive facets for the radius, radiale, and distally, for the distal carpal 2 + 3. Distal carpals 1 and 2 + 3 only preserve their posterior portion. Distal blocks of the forelimb preserve indeterminate bony elements as well as phalanges, which are elongated and dorsoventrally flattened.

**Figure 11:**
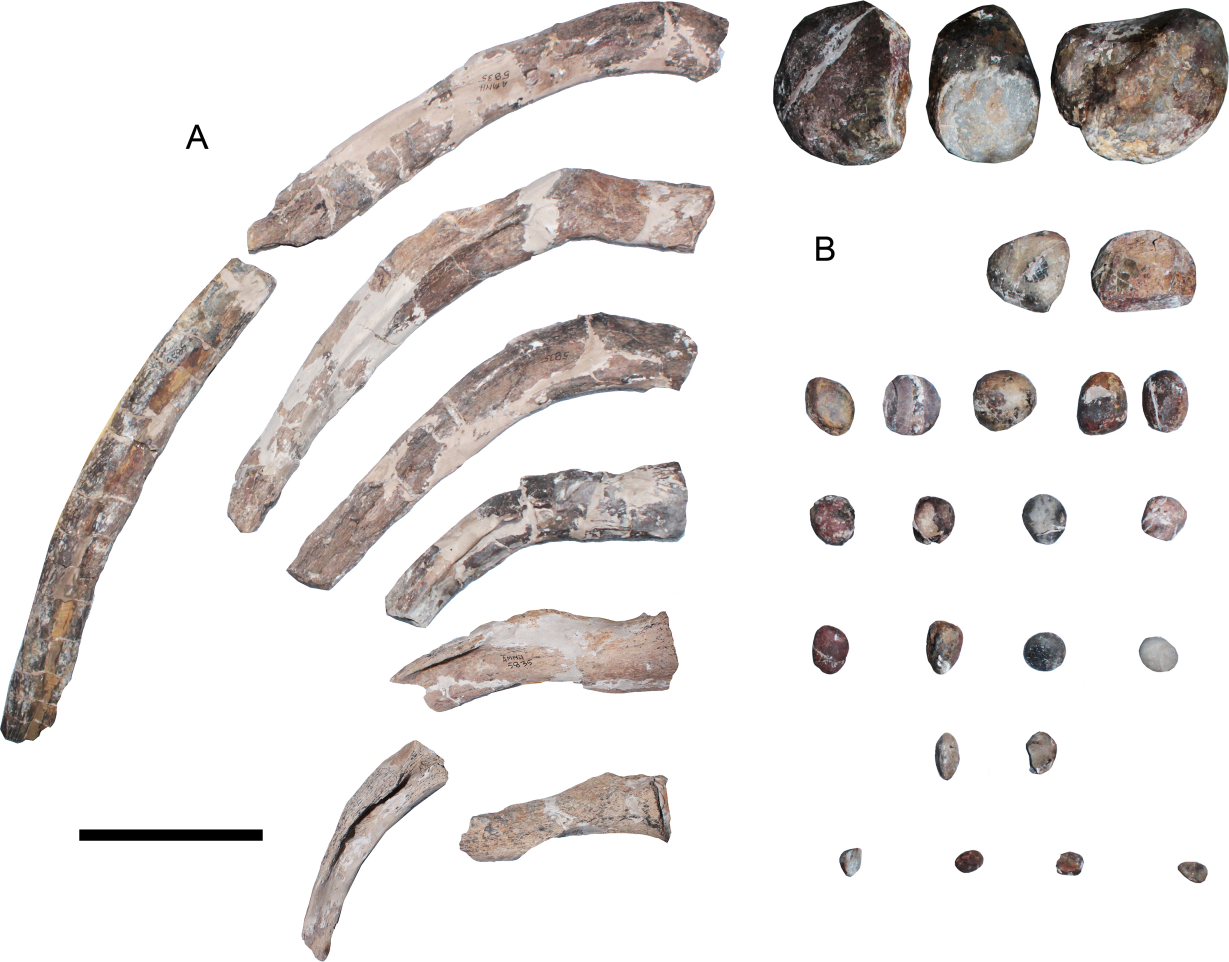
*Styxosaurus browni* (Welles, 1952) (AMNH 5835). Ribs and gastroliths. (A) assorted dorsal ribs. (B) gastroliths associated to the skeleton. Scale bar equals 10 cm.

**Ribs**—Isolated ribs are found among the material (Fig. 11A). The longest elements, likely from the central part of the trunk, have a rounded to oval cross-section, while the posterior dorsal ribs have a posterodorsal keel that turns into a sharp ridge. These ribs are proximally straight (horizontal) and medially, they become dorsally convex.

**Gastroliths**—Twenty-four gastroliths are associated to the AMNH 5835 (Fig. 11B). Their size varies from almost decimetric clasts to smaller rocks around 10 mm. Larger clasts are subrounded, with some edges still prominent. Smaller clasts are rounded and few of them are oval. There is no taphonomic information regarding the anatomical position of these elements in the fossil.

## Discussion

**Anatomical reassessment of AMNH 1495 elements**—58 cervical vertebrae, fifteen dorsal vertebrae, three sacral vertebrae and twenty caudal vertebrae of AMNH 1495 were found, adding up to a total of 98 vertebrae. Welles (1952: p. 61) indicated a total of 104 vertebrae for this specimen, plus 7 probably missing. Thus, 6 centra seem to be lost. The three pectorals and three anterior dorsals here reported as missing could potentially account for the extra vertebrae reported by Welles (1952). The total number of cervical vertebrae cannot be determined. Welles (1952) reported that at least one anterior cervical vertebra (aside from the atlas-axis) is missing. Also, posterior cervicals are unordered and unnumbered; a few of them are partially covered by the matrix or else are fragmentary. Finally, the pectoral vertebrae are missing. This makes it difficult to evaluate the continuity of the axial skeleton between the neck and the trunk. AMNH 1495 possesses 58 or more cervical vertebrae, but the precise number is uncertain.

The two preserved propodials of AMNH 1495 are here identified as the femora, while the elements of the forelimb previously described by Watson (1924), Welles (1943: Fig. 29) and Welles (1952: Fig. 4B) were not found among the material. Welles (1952: Fig. 4B) described the forelimb of AMNH 1495 being composed of: a humerus articulated with the radius, a partial ulna and the radiale. In the first description of ‘*Hydralmosaurus*’ (Welles, 1943: Fig. 29) the humerus of that forelimb appears with a postaxial distal margin shorter than that illustrated later, in 1952. Among the schemes of AMNH 5835, holotype of *Styxosaurus browni* (Welles, 1952: Fig. 7), a remarkably similar forelimb was displayed, which preserves precisely the same elements (humerus, radius and radiale) described for AMNH 1495, even with the very same damaged margins. The humeri in both images (Welles, 1952: Figs. 4B and 7) have a sigmoidal shaft and a remarkably extended postaxial distal margin. In the first mention of AMNH 1495, Cope (1877: p. 580) states the following: “The anterior limbs are a little the larger. The humerus is very robust; its shaft is subcylindric, and the distal extremity is greatly expanded, so that the width is but little less than the length. The proximal end of the shaft continues in a plane without curvature, which terminates in a broadly truncate tuberosity with prominent lateral ridges”. This description explicitly indicates that the shaft is straight (“without curvature”). Considering this, the outline proposed by Welles (1952: Fig. 4B) was likely confused with the forelimb of AMNH 5835.

Regarding the pelvic girdle, the outline of the pubis cannot be verified on the grounds of the available material.

**Taxonomical reassessment of AMNH 1495**—The first distinction of AMNH 1495 from other plesiosaurians was proposed by Cope (1877: p. 578, 579). During that time, the only plesiosaurian with closer affinities to AMNH 1495 was ANSP 10081 (type of *Elasmosaurus platyurus*). Cope noted several differences between the cervical vertebrae of both specimens, most of them regarding their proportions among equivalent elements of the neck. AMNH 1495 had shorter cervical vertebrae; this specimen also lacked a “longitudinal lateral angle” (=lateral keel) in the last eighteen cervical vertebrae. Based on this, Cope erected a new species, ‘*Elasmosaurus serpentinus*’ (Cope, 1877). Welles (1943) erected a new genus, *Hydralmosaurus*, considering AMNH 1495 as its type species and proposing the new combination *H. serpentinus*. Diagnostic features of this genus and species included the absence of pectoral and pelvic bars, the absence of a lateral keel on posterior cervical vertebrae, the presence of cordiform fenestra on the coracoids, pubes with a concave anterior border, a humeral head well separated from the tuberosity, and a well developed epipodial foramen. The absence of pectoral and pelvic bars is a common feature present in many adult elasmosaurids worldwide (Hector, 1874; Hutton, 1893; Colbert, 1949; Welles, 1943; Welles, 1952; Welles, 1962; Hiller et al., 2005; Otero, Soto-Acuña & Rubilar-Rogers, 2012; Otero et al., 2014c). A similar case occurs among the pelvic girdles of elasmosaurids. Due to this, the absence of pectoral and pelvic bars in AMNH 1495 cannot be considered as diagnostic to genus or species level.

On the other hand, the presence of lateral keels on the cervical vertebrae is a variable feature within elasmosaurids. It may vary among taxa, but the real diagnostic value of this character needs to be established on the grounds of complete necks of adult individuals, because this feature varies during ontogeny and along the neck. In addition, the presence of a humeral head well separated from the tuberosity is a feature also present in many elasmosaurids worldwide (Welles, 1962; Sato, 2003; Hiller et al., 2005; O’Gorman, Gasparini & Salgado, 2013; Otero, O’Gorman & Hiller, 2015) and should be rejected as diagnostic. A similar case happens with the epipodial foramen, present in many elasmosaurids (*S. browni*, *C. colombiensis, Morenosaurus stocki,* SDSM 451, among others) but absent in others as well (CM Zfr 115, *Aristonectes parvidens*, *Aristonectes quiriquinensis*, among others). Thus, such feature can be taxonomically useful but it is not diagnostic to genus level. Then, the combination of characters proposed by Welles (1943) can be found in various elasmosaurids. This is the reason why a new set of diagnostic characters is here presented (see ‘Systematic Paleontology’).

In addition, Carpenter (1999) considered the presence of 63 cervical vertebrae on AMNH 1495 (here reduced to at least 58 verified cervical vertebrae) as a diagnostic feature of the species ‘*Hydralmosaurus serpentinus*’, separating it from other elasmosaurids such as *S. snowii*, considered to have 62 cervical vertebrae based on referred specimens but having only 28 in the holotype. He also considered as diagnostic features a humerus with a pronounced posterior expansion on its distal end (only verifiable on AMNH 5835 and missing on AMNH 1495), and the presence of pectoral and pelvic bars, a feature widely present in known elasmosaurids. Thus, all the features considered by Carpenter (1999) cannot be currently considered as diagnostic to genus nor species level.

The hindlimb of AMNH 1495 is unique among known elasmosaurids, due to the presence of a fibula with a deep concave postaxial margin, likely for accommodating a sesamoid element, along with the presence of a sesamoid fused to the postaxial margin of the fibulare. Although the latter feature could occur among other elasmosaurids, this is the first documented case where both elements are fused but still clearly visible. The presence of a sesamoid settled in an intermediate position between the fibula and the fibulare, as well as a second sesamoid related only to the fibulare, also occurs in the forelimb of *Terminonatator pointeixensis* (Sato, 2002: Fig. 2.5; Sato, 2003: Fig. 13B). However, in this taxon the distal sesamoid is not fused to its ulnare, as it occurs in the fibulare of AMNH 1495. Additionally, the ilium is unique among known elasmosaurids for having a triangular outline with a recurved dorsal facet. The cervical proportions are useful for matching AMNH 1495 with the holotype of *S. snowii*, as well as with the holotype of *S. browni*. Finally, it must be addressed that the proportions of equivalent cervical elements are remarkably similar between the three specimens (Table 5). However, AMNH 1495 does not preserve enough diagnostic characters for a specific determination, and for that reason it is here referred as *Styxosaurus* sp.

**Table 5:** Comparative measurements of equivalent cervical vertebrae. Measurements of AMNH 1495, AMNH 5835, ANSP 10081, KUVP 1301 and CM Zfr 115 are presented. Information of ANSP 10081 and KUVP 1301 taken from Welles (1952).

Numeration on specimen	AMNH 1495	KUVP 1301	AMNH 5835	ANSP 10081	CM Zfr 115
**Length**					
1				30	25.75
2			38.46	29	29.84
3	39.67	23	41.93	39	31.5
4	41.42	30	45.42	42	32.9
6	44.84	48	49.56	43	35.64
9	53.12	53	53.91	50	36.85
14	56.96	63	64.36	55	43.14
20	79.03	78	70.97	67	54.04
27	94.09	90	87.35	83	61.21
**Height**					
1	–	25	–	27	24.36
2	–	27	29.77	25	25.03
3	35.15	–	28.36	25	26.92
4	34.78	–	33.65	27	26.94
6	37.08	42	32.77	30	30.21
9	41.65	44	30.55	–	32.33
14	39.52	50	37.55	35	52.22
20	56.84	60	42*	42	42.36
27	63.75	68	–	46	49.88

**Re-validation of *Styxosaurus browni* (Welles, 1943) (AMNH 5835)**—As pointed in the emended diagnosis of AMNH 5835, this specimen could be included within the genus *Styxosaurus*, based on the close morphological affinities with the skull and cervical vertebrae of KUVP 1301 (holotype of *S. snowii*). However, slight differences between both skulls justify their separation as different species. Cranial differences include the presence of a jugal bar higher than that of *S. snowii*, a higher temporal ridge, a preorbital boss rounder and larger than that of *S. snowii*, a dorsal contact of squamosals not prominent with respect to the sagittal crest (which indeed occurs on *S. snowii*), and finally, the height of posterior process of the maxilla reaches the half of the jugal bar (contrary to *S. snowii* where this is one third the height of the jugal bar). On the other hand, comparison of the *S. browni* holotype with AMNH 1495 shows differences on the axial skeleton and mostly in the pectoral girdle. AMNH 1495 possesses a shortened centrum c35, here interpreted as a cervical node, which is absent on AMNH 5835. Also, anterior dorsal vertebrae of AMNH 1495 have transverse processes recurved dorsally in an angle between 30°and 35°, while in AMNH 5835 anterior dorsal vertebrae are almost horizontal, becoming slightly recurved dorsally (but below 30°) from middle dorsal vertebrae and backwards. Pectoral girdles differ at least in the scapula and coracoids. The AMNH 5835 scapula has a glenoid portion thicker than that of AMNH 1495, with articular facets more defined (the latter could be ontogenetic). Also, the scapular shaft is shorter on AMNH 5835 with respect to AMNH 1495. Coracoids are also different. AMNH 1495 has mid anterior processes comparatively larger and more recurved than those of AMNH 5835. On the other hand, the AMNH 5835 humerus appears as having a sigmoidal shaft, however, most of its medial portion is reconstructed, making this character at least questionable.

Differences between AMNH 5835 and KUVP 1301 have been previously addressed, mostly on the grounds of skull comparisons. Welles (1952) separated both skulls based on the presence of thirteen maxillary teeth on AMNH 5835 and fifteen on KUVP 1301.This author also considered the skull length, being 37 cm on AMNH 5835 and 42 cm on KUVP 1301, however, the snout and dentaries of the first are incomplete, and therefore, the complete skull length is a value closer to that of KUVP 1301. Welles (1952) also used the ‘beak index’ (42 on KUVP 1301 and 39 on AMNH 5835), defined as the ratio of the distance between the anterior border of the premaxillary to the anterior orbital border, with respect to the total length of the skull measured from the anterior border of the premaxilla until the occipital condyle. Again, this minor difference could be caused by the incomplete rostrum of AMNH 5835. Also, the maxillary tooth count difference (thirteen on AMNH 5835 versus fifteen on KUVP 1301) could reflect ontogenetic differences, loss of teeth by mechanical removal, or replacement of dental pieces. Thus, no actual autapomorphic skull features were recognized at that time between both specimens.

Carpenter (1999) referred AMNH 5835 to the species ‘*Hydralmosaurus serpentinus*’ following the diagnostic features explained above. AMNH 5835 possesses 62 cervical vertebrae (numbered c2–c63 on the specimen). Indeed, 62 cervical vertebrae were regarded by the same author as a character present in *Libonectes morgani*, *Thalassomedon haningtoni* and *Styxosaurus snowii* (despite the fact that the holotype of the latter only preserves 28 cervical vertebrae). Once again, the real diagnostic value of the cervical count should be evaluated among specimens with complete necks. This is not the case of AMNH 5835; the posterior cervical vertebrae are difficult to interpretate and a few of them remain unnumbered. This could easily cause a difference in one cervical. Other diagnostic characters considered by Carpenter (1999) are the absence of pectoral and pelvic bar (discussed above as being the condition in most Late Cretaceous elasmosaurids) and the pronounced expansion of the humerus posterodistal margin. This latter character is indeed present on AMNH 5835.

Summarizing, it is difficult to hold a generic separation on the sole ground of a differentially elongated distal postaxial margin of the humerus. Such feature could even change throughout its ontongeny. Also, the real sigmoidal profile of AMNH 5835 humerus is at least questionable because a good deal of the shaft has been reconstructed.

**Figure 12:**
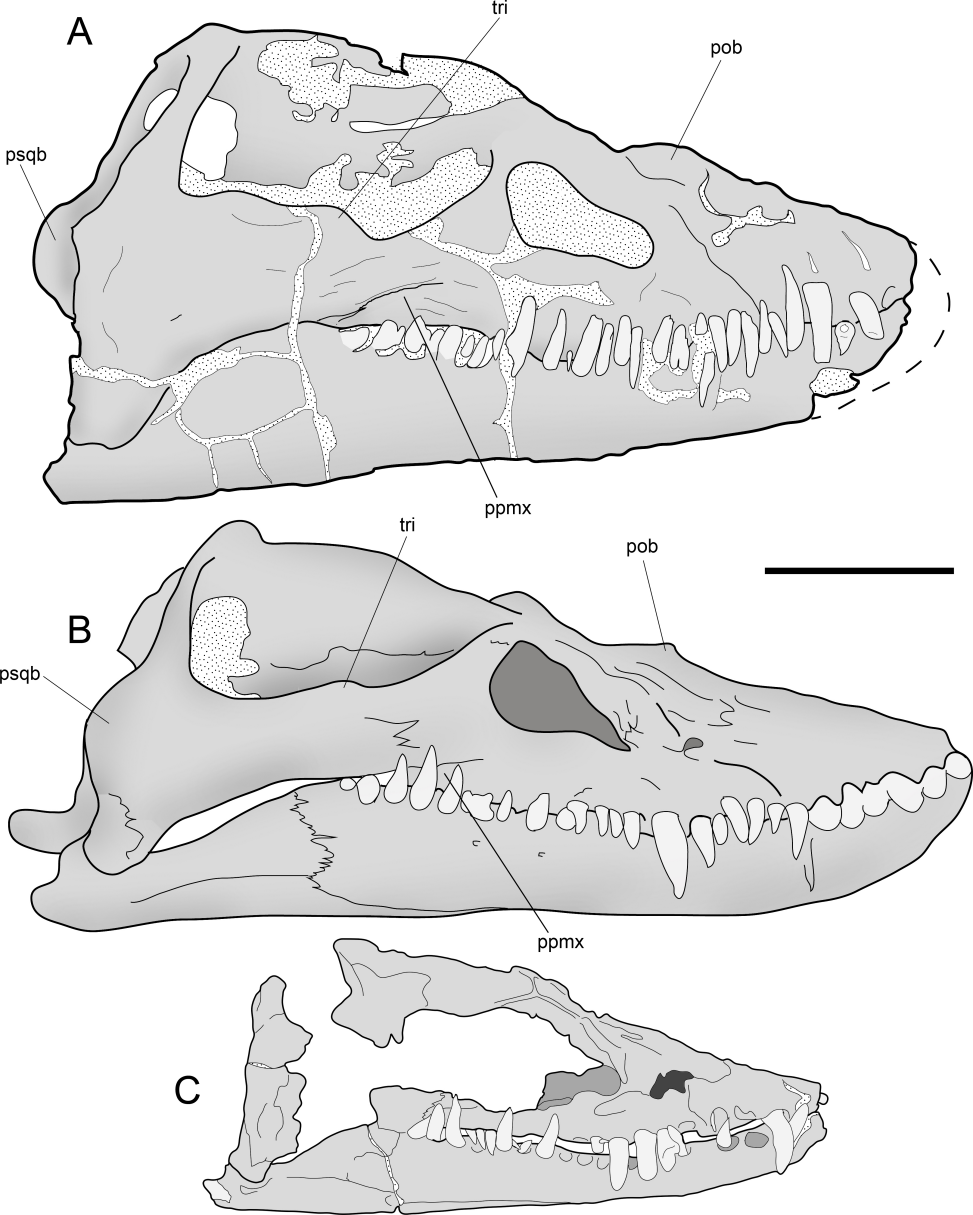
Schematic comparison of styxosaurine skulls. (A) *Styxosaurus browni* (AMNH 5835, holotype). (B) *Styxosaurus snowii* (KUVP 1301). (C) *Terminonatator pointeixensis* (RSM P2414.1, holotype). Anatomical abbreviations: pob, preorbital bulk; ppmx, posterior process of the maxillar; psqb, posterior squamosal bulk; tri, temporal ridge. Scale bar equals 10 cm.

On the other hand, there are evident similarities between the skulls of AMNH 5835 and KUVP 1301. Despite the fact that AMNH 5835 is laterally crushed and KUVP 1301 is dorsoventrally compressed, both have congruent lengths and possess a similar reniform orbit. In the case of KUVP 1301, the latter is dorsally collapsed, deforming the dorsal outline. This was misinterpreted by Carpenter (1999: Fig. 10) who considered the orbit as being almost rounded, with the external naris placed immediately anterior to the latter. Both structures are indeed the orbit, while the external naris of KUVP 1301 is obscured by the crushing and is placed likely near the maxillary-premaxillary suture. Another common feature in both skulls is the presence of a posterior boss on the squamosal. Carpenter (1999: Fig. 7) described the squamosal as being posteriorly straight. This prominent process is broken on the right squamosal of AMNH 5835, however, it is visible on their left one, being very similar to that observed on KUVP 1301. Also, both skulls possess a singular structure on the dorsal margin of the temporal bar, here nominated as temporal ridge, that is higher on AMNH 5835. Another interesting feature is the common presence of a preorbital boss in both skulls, although, it is not clear if this could be taphonomic or not. A scheme of both skulls is provided (Fig. 12) and complemented with a third taxon here included in the Styxosaurinae, *Terminonatator pointeixensis*. The scheme shows general affinities between these taxa despite their evident differences in size. It must be noted that the preorbital boss in the latter is not observed.

**Phylogenetic analysis**—Analysis of the dataset of Benson & Druckenmiller (2014), plus the thirteen additional elasmosaurid OTUs here added, was first performed using implied weighting (K = 3; New Technology Search, Tree Fusing). This returned one most parsimonious cladogram (CI = 0.27; RI = 0.68; 2,511 steps). All the taxa here referred to the Styxosaurinae were recovered in a single branch (Data S1, Fig. 1). A second analysis considered Bootstrap support (2000 replicates, Standard, New Tech Search, Tree fusing) which was applied to the same datamatrix. This returned good stability for the Styxosaurinae (Data S1, Fig. 2). The congenerity of AMNH 1495, AMNH 5835 and *Styxosaurus snowii* is well supported too. The clade Elasmosauridae was returned as unstable.

**Figure 13:**
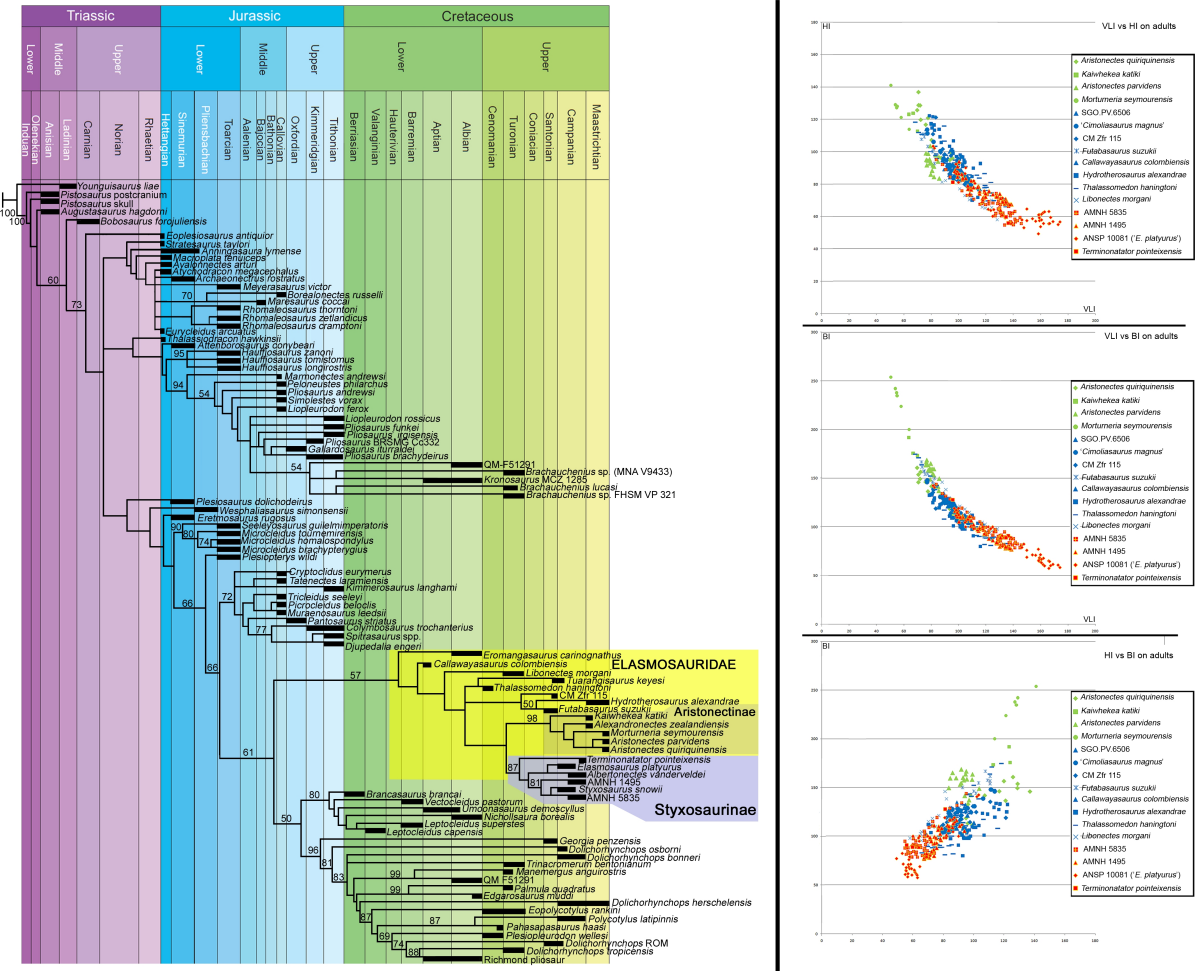
Hypothesis of relationships of the Elasmosauridae and plots obtained with the bivariate analysis. (A) Time-callibrated cladogram based on the datamatrix of Benson & Druckenmiller (2014) with the modifications introduced here. Phylogenetic result is based on Data S1, Fig. 3. Bootstrap values over 50% are indicated on each branch. (B) Plots of adult elasmosaurids. Green marks are different aristonectine taxa. Blue marks represent elasmosaurids with ‘*Cimoliasaurus*’-grade cervicals. Finally, red marks represent different taxa within the Styxosaurinae.

A third analysis pruned the fragmentary elasmosaurid *Wapuskanectes betsynichollsae* (86.7% of missing data) and the Speeton Clay Plesiosaurian (63.3% of missing data). On the other hand, the specimen GWWU.A3.B2, returned within the Elasmosauridae by Benson & Druckenmiller (2014), was described in detail by Hampe (2013) who identified as a new genus and species, *Gronausaurus wegneri*. This taxon shows affinities to *Brancasaurus brancai*, and it was subsequently referred to the Leptocleidia. Thus, it is evident that the phylogenetical position of this specimen needs to be revised. Due to this, this taxon was pruned from subsequent analysis. In addition, few unstable taxa detected in preliminary resamplings and within the Cryptoclidia were pruned. These are ‘*Cimoliasaurus*’ *valdensis*, ‘*Plesiosaurus*’ *mansellii*, *Abyssosaurus nataliae* and Speeton Clay Plesiosaurian. As a result of excluding these six taxa, several clades within the Cryptoclidia were returned with enough support. The clades Elasmosauridae (57%), Leptocleididae (80%), Polycotylidae (98%) and Cryptoclididae (72%) were returned with enough stability. The clade Styxosaurinae returned 87% of support. Congenerity of AMNH 5835, AMNH 1495 and *Styxosaurus snowii* returned 81% (Data S1, Fig. 3). Based on the last analysis, a time-callibrated cladogram is proposed (Fig. 13A).

**Bivariate analysis**—Plotting of adult or near adult elasmosaurids from the Weddellian Biogeographic Province, the Western Interior Seaway and a single sub-equatorial taxon (*C. colombiensis*) returned interesting patterns. Graphic relationships of VLI versus HI, VLI versus BI, and HI versus BI (Fig. 13B) show disparate neck types among aristonectines and styxosaurines in all cases. The first are characterized by low VLI and high HI and BI values, reflecting the possession of cervical centra shortened with respect to all other elasmosaurids. On the other hand, styxosaurines display the highest VLI and low BI and HI values, thus, reflecting that their cervical vertebrae include the longest centra among elasmosaurids. Such disparate vertebral types only occur in partial segments of the neck. Among aristonectines, *Aristonectes quiriquinensis* has shortened cervical vertebrae between c27 and c30; *Aristonectes parvidens* shows shortened centra on c2, c6, c7, c11 and c18; *Kaiwhekea katiki* has shortest centra on c5–c7, however, VLI values for the rest of the neck are unavailable because the state of preservation. This made the evaluation of the breadth of most centra difficult. All the cervical vertebrae of *Morturneria seymourensis* have a disparate plotting, but their position along the neck is uncertain. In the case of styxosaurines, the most elongated cervical vertebrae of *Styxosaurus browni* are c11–c20; *Elasmosaurus platyurus* has its longest cervical vertebrae at least between c24 and c47; in the case of AMNH 1495 its longest cervical vertebrae are between c23 and c30. These values evidence that even among closely related forms, cervical elongation or shortening occurred in variable amounts and among different cervical elements. It is possible that such variation could be taxonomically helpful, however, such comparison should be attempted only between complete necks.

An interesting pattern is visualized in the plotting of AMNH 2554 (type of ‘*Cimoliasaurus magnus*’), CM Zfr 115, *Callawayasaurus colombiensis*, *Hydrotherosaurus alexandrae*, SGO.PV.6506 and *Thalassomedon haningtoni*. All these specimens share cervical vertebrae with a low dispersion (distance between plotted points of a single taxon), indicating that variation among cervical elements (elongation or shortening) is not as marked as in styxosaurines or aristonectines. This suggests that such kind of vertebrae could represent a plesiomorphic condition from which both disparate groups derived. In this sense, Otero et al. (2015a) proposed an informal group for reuniting specimens possessing such kind of cervical vertebrae, naming it ‘plesiomorphic elasmosaurids’ in order to distinguish them from aristonectines and from ‘extremely long-necked elasmosaurids’. These authors recognized a separation of these three groups among juvenile and especially among adult individuals.

**Statistical testing of the cervical morphological segregation**—The Kruskal-Wallis test returned the probality of the null hypothesis between comparisons of dataset pairs (the Aristonectinae, the Styxosaurinae and the ‘*Cimoliasaurus*’-grade morphotype). This test was significant for the pair comparisons (*p* = 8.216*E* − 51). As a result, the following values were obtained (Bonferroni corrected): ‘*Cimoliasaurus*’-grade morphotype v/s Aristonectinae: *p* = 6.709*E* − 17; ‘*Cimoliasaurus*’-grade morphotype v/s Styxosaurinae: *p* = 5.914*E* − 38; finally, Aristonectinae v/s Styxosaurinae: *p* = 3.805*E* − 22. These significant values point out that the null hyphotesis is improbable.

**Figure 14:**
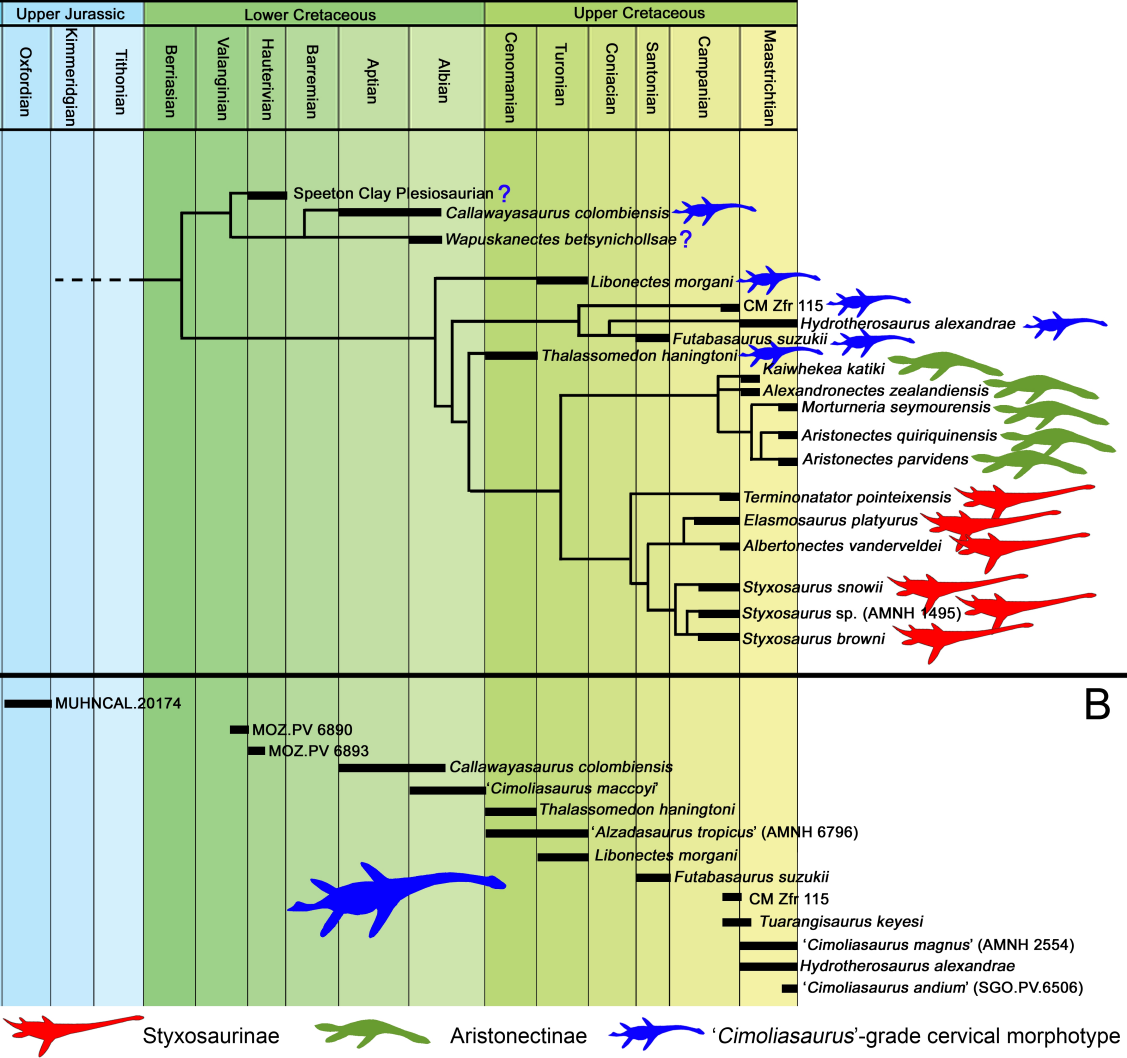
Elasmosaurid morphotypes through time. (A) Phylogenetic-based tree of the Elasmosauridae calibrated through time. Two Late Cretaceous groups, the Aristonectinae and the Styxosaurinae, are clearly distinguished. (B) Chronostratigraphic occurrence of elasmosaurids with ‘*Cimoliasaurus*’-grade cervical morphotype. This occurs persistently throughout the Cretaceous and is present even among Upper Jurassic specimens.

**Cervical vertebral morphotypes within the Elasmosauridae**—This study only considered adult specimens with fairly complete necks, aiming to avoid changes due to different ontogenetic stages. Intracolumn variation was treated in the bivariate analysis, identifying the most disparate elements on each taxon. Finally, in order to help to the taxonomic variation, and based on the information exposed above, this research proposes the ‘*Cimoliasaurus*’-grade cervical morphotype. This is not a valid taxonomical group; indeed, it is clearly paraphyletic. Such separation, based on cervical proportions, was noted even in the early years of vertebrate paleontology by Cope (1868) and Leidy (1870a; Leidy, 1870b). Such cervical vertebrae were frequently discussed during the 20th century (Welles, 1943; Welles, 1952; Welles, 1962; Persson, 1960; Persson, 1963). This morphotype even led to the coining of a new family of plesiosaurians, the ‘Cimoliasauridae’ (DeLair, 1959). This morphotype is also coincident with the concept of non-elongate taxa of O’Keefe & Hiller (2006) and with the ‘plesiomorphic’ elasmosaurids proposed by Otero et al. (2015a). Following O’Keefe & Street (2009), it is clear that ‘Cimoliasauridae’ is a junior synonym of Elasmosauridae, while ‘*Cimoliasaurus magnus*’ is a void name and its type specimen, AMNH 2554, an indeterminate elasmosaurid. Cervical vertebrae with similar proportions have been frequently found throughout the whole Cretaceous and distributed worldwide. This ‘*Cimoliasaurus*’-grade cervical morphotype indeed occurred associated to Campanian styxosaurines on the Western Interior Seaway, and was found associated to Maastrichtian aristonectines on the Weddellian Biogeographic Province. The phylogenetic hypothesis here obtained shows that different taxa of elasmosaurids with ‘*Cimoliasaurus*’-grade cervical vertebrae are sister taxa of the styxosaurines and the aristonectines. Furthermore, this cervical morphotype is already present on basal elasmosaurids (Fig. 14A), however, the relationships between different taxa other than styxosaurines and aristonectines are still unclear. Interestingly, the oldest plesiosaurian records with ‘*Cimoliasaurus*’-grade cervical vertebrae are mostly restricted to the Southern Hemisphere during the Lower Cretaceous (Kear, 2002; Lazo & Cichowolski, 2003; O’Gorman et al., 2015a), with one known exception in the upper Hauterivian of Germany (Sachs et al., 2015). This reached an sub-equatorial distribution during the ‘middle’ Cretaceous (Welles, 1962; Carpenter, 1999) and acquired a worldwide distribution during the Late Cretaceous (Fig. 14B), being present until the K/Pg boundary (Hiller et al., 2005; Otero et al., 2014a). Furthermore, an articulated cervical series from Oxfordian beds of northern Chile (MUHNCAL.20174) has similar morphologies and cervical indices and it could represent the oldest known elasmosaurid (Otero et al., 2015b).

**Figure 15:**
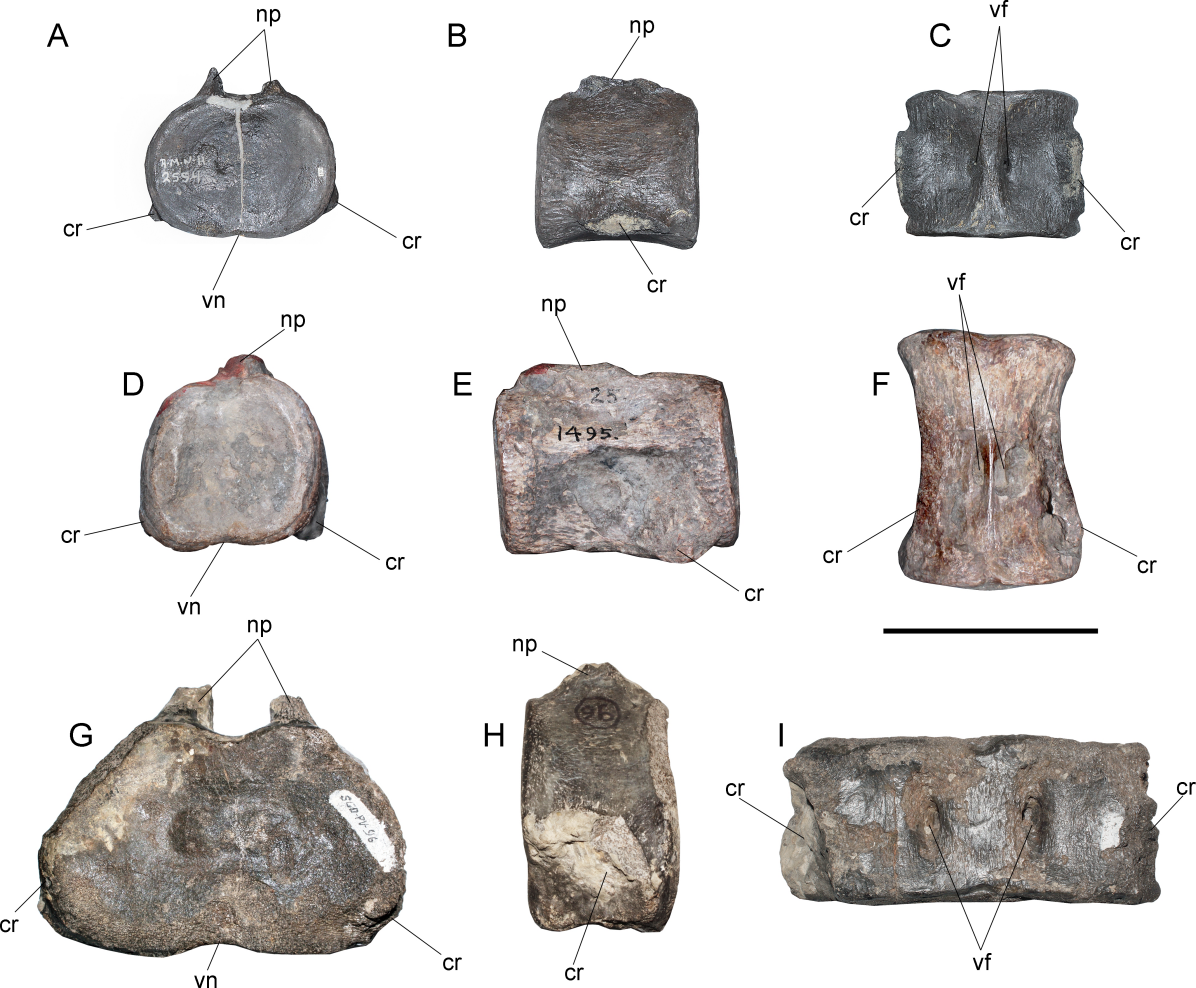
Three main cervical morphotypes among elasmosaurids. ‘*Cimoliasaurus*’-grade cervical centrum, represented by a selected cervical of AMNH 2554, type specimen of ‘*Cimoliasaurus magnus*’ (*nomen vanum*) from the Hornerstown Formation of New Jersey (Maastrichtian). (A) anterior view. (B) left lateral view. (C) ventral view. ‘Can-shaped’ cervical type, typical of Styxosaurines and represented by the cervical c25 of AMNH 1495 referred to *Styxosaurus* sp. from the Pierre Shale group, central USA (middle to upper Campanian). (D) anterior view. (E) left lateral view. (F) ventral view. Aristonectine type represented by the isolated cervical SGO.PV.96 (Quiriquina Formation of central Chile, upper Maastrichtian). (G) anterior view. (H) left lateral view. (I) ventral view. Anatomical abbreviations: cr, cervical ribs; np, neural pedicels; vf, ventral foramina; vn, ventral notch. Scale bar equals 10 cm.

As a result, three main morphotypes of cervical vertebrae are recognized among elasmosaurids. (i) ‘*Cimoliasaurus*’-grade cervical vertebrae, explained above, characterized by centra similarly larger than high and broader than long or high (Fig. 15A). This type occurs on all or most of the cervical vertebrae of a single individual; (ii) “can-shaped” cervical vertebrae. This follows the concept of ‘elongate-taxa’ by O’Keefe & Hiller (2006) and the extreme long-necked elasmosaurids by Otero et al. (2015a). These are disparate elements frequently occurring in the mid-part of the neck, having centra almost twice as long as they are high and with heights similar to their breadth (Fig. 15B). These cervical vertebrae are typical of styxosaurines; finally, (iii) the aristonectine-type cervical vertebrae, which are also disparate elements drastically shortened, indistinctly occurring in the anterior, middle or posterior part of the neck and likely varying between aristonectine taxa. This type of cervical vertebrae conforms most of the neck in adult aristonectines, however, they can eventually occur in a few cervical vertebrae of immature or young elasmosaurids with ‘*Cimoliasaurus*’-grade proportions (i.e., AMNH 5621, the very juvenile holotype of ‘*Leurospondylus ultimus*’ Brown, 1913). Thus, suggesting a paedomorphic origin of the aristonectines. These are characterized by centra about twice as broad as they are long and higher than they are long (Fig. 15C). It must be stated that among cervical vertebrae of both styxosaurines and aristonectines, some cervical centra can possess proportions similar to ‘*Cimoliasaurus*’-grade cervical vertebrae. This is evident in the bivariate analysis, and because of it, the morphological distinction of these three cervical types considers only disparate centra. The transverse presence of ‘*Cimoliasaurus*’-grade cervical vertebrae among basal elasmosaurids as well as in the clades Styxosaurinae and Aristonectinae strongly supports this cervical type as a plesiomorphic feature, which was variably retained in different sections of the neck of different elasmosaurid taxa. In addition, fairly complete individuals possesing exclusively ‘*Cimoliasaurus*’-grade cervical vertebrae without disparate elements are present since the Upper Jurassic (e.g., MUHNCAL.20174) until the Maastrichtian (e.g., CM Zfr 115, *Hydrotherosaurus alexandrae*). This indicates that the most common cervical type among elasmosaurids is indeed the ‘*Cimoliasaurus*’-grade morphotype, while disparate styxosaurine- and aristonectine-types are derived features acquired separately in representatives with biogeographical affinities.

**Axial formula**—The real number of cervical vertebrae among elasmosaurids from the Western Interior Seaway has been discussed above, however, most of the diagnostic specimens possess fairly complete necks with few centra that could be missing in each case. Thus, the following analysis regarding their axial formulae is not absolute in terms of cervical count, but it is informative in terms of general axial changes. Table 6 summarizes the vertebral count of different plesiosaurians with fairly complete skeletons. As a first approach, it is evident that neck elongation occurred among different plesiosaurian lineages in different lapses. Lower Jurassic microcleidids such as *Microcleidus tournemirensis* (Sciau, Crochet & Mattei, 1990) acquired long necks with 41 cervical vertebrae. However, the pectoral and dorsal number on this species is similar to those found in derived lineages. Upper Jurassic-Lower Cretaceous cryptoclidids also have an event of neck elongation represented by *Spitrasaurus* spp., reaching the remarkable number of 60 cervical vertebrae, a character classically regarded only to elasmosaurids (Carpenter, 1999). Even among polycotylids events of neck elongation existed, evidenced by *Thililua longicollis* Bardet, Pereda-Suberbiola & Jalil, 2003, which has 30 cervical vertebrae. Thus, besides Elasmosauridae, neck elongation occurred at least three times and at least in three different clades (microcleidids, cryptoclidids and polycotylids).

**Table 6:** Axial formulae of different plesiosaurians. Axial sections with known missing centra are denoted with a (+) following the number of preserved centra.

Taxon	Cervicals	Pectorals	Dorsals	Sacrals	Caudals	Source of information
*Microcleidus tournemirensis*	41	3	16	4	–	Bardet, Godefroit & Sciau, 1999
*Cryptoclidus eurymerus*	29–32	3	20–23	–	–	Brown, 1981
*Spitrasaurus* spp.	60	3	–	–	–	Knutsen, Druckenmiller & Hurum, 2012a; Knutsen, Druckenmiller & Hurum, 2012b
*Nichollssaura borealis*	24	3	22	4	28–29	Druckenmiller & Russell, 2008
*Brancasaurus brancai*	32	3	22	3	26	Wegner, 1914
Speeton Clay plesiosaurian	23+	3	19–20	3	17+	Review from photographies
*Futabasaurus suzukii*	26+	3	18	4	–	Sato, Hasegawa & Manabe, 2006
*Callawayasaurus colombiensis*	56	2	23	–	–	Welles, 1962
*Libonectes morgani*	50–62	–	–	–	–	Welles, 1949
*Libonectes atlasense*	52–53	5	16–17	–	–	Buchy, 2005
*Aristonectes quiriquinensis*	43	3	23–24	3	35	Otero et al., 2014c
*Kaiwhekea katiki*	43	3	20	–	–	Direct review, 2013
*Hydrotherosaurus alexandrae*	60	2	17	3	20	Welles, 1943
CM Zfr 115	63	2	18	–	20+	Direct review, 2013
*Elasmosaurus platyurus*	72	5	18	6	21+	Sachs, 2005
*Thalassomedon haningtoni*	62	3	25	3	21	Welles, 1943
*Styxosaurus browni* (AMNH 5835)	62+	–	11+	–	–	Direct review, 2015
*Styxosaurus snowii* (KUVP 1301)	28+	–	–	–	–	Williston, 1890; Welles, 1952
*Styxosaurus* sp. (AMNH 1495)	58+	3	19	3	22+	Direct review, 2015
*Albertonectes vanderveldei*	76	?	18	5	33	Kubo, Mitchell & Henderson, 2012
*Trinacromerum bentonianum*?	19	3	15+	3	–	Williston, 1903
*Terminonatator pointeixensis*	51+	?	17	4	12+	Sato, 2003
*Tililua longicollis*	30	3	4+	–	–	Bardet, Pereda-Suberbiola & Jalil, 2003

Within the clade Elasmosauridae, the drastic neck elongation is represented by styxosaurines, but it is also present in representatives with ‘*Cimoliasaurus*’-grade cervical vertebrae possessing cervical counts greater than 50. This points to at least two different events for elasmosaurid neck elongation. A possible third event might be represented by the Speeton Clay plesiosaurian, which preserves only 23 cervical vertebrae. However, these have proportions remarkably similar to styxosaurines. Considering these (at least) three events within Elasmosauridae, the evolutionary history of plesiosaurians has at least six documented events of neck elongation, and at least one event of neck shortening, the latter derived from ancestors with long necks, as is the case of aristonectines.

The pectoral and caudal counts of elasmosaurids seem to be scarcely modified and vary between three to five on each case. However, dorsal count among elasmosaurids varies between 17 and 25. Most basal and also complete elasmosaurid skeletons such *Callawayasaurus colombiensis* and*Thalassomedon haningtoni* have 23 and 25 dorsal vertebrae, respectively. On derived elasmosaurids, this number is reduced to 17–19. Such number is present on *Futabasaurus suzukii*, *Hydrotherosaurus alexandrae*, *Elasmosaurus platyurus*, *Albertonectes vanderveldei*, AMNH 1495, CM Zfr 115, and *Terminonatator pointeixensis*. With the exception of *Futabasaurus* which has an incomplete neck, all these specimens show a high number of cervical vertebrae coupled with a reduction of the plesiomorphic dorsal count from 23–25 to 17–19 centra. Among the latter taxa, with the exception of the unknown total of *F. suzukii*, all of them possess at least 51 cervical vertebrae. Based on the studied axial formulae (Table 6), a variable estimation of five to eight post-cervical centra are added to the neck in derived elasmosaurids. This evidence also supports that the highest cervical counts are not only the result of this homeotic shifting, but also a result of the acquisition of additional cervical centra.

**Table 7:** Effective neck length among elasmosaurids. The total length of each neck is compared. Cervical numbers with estimated additional centra are noted with (+).

	CM Zfr 115	*Libonectes morgani*	AMNH 1495	*Thalassomedon haningtoni*	AMNH 5835	*Elasmosaurus platyurus*	*Hydrotherosaurus alexandrae*	*Albertonectes vanderveldei*
Cervicals with unavailable length	5	3	5	1	3	3	1	–
Average length of cervical centra	61,85	84,42	89,11	99,12	88,26	87,03	75,14	–
Total length of available centra	3587,48	4812	4723,22	6046,12	5207,12	5918	4283	–
Total estimated length (replacing missing centra with the average length value)	3896,74	5065,26	5168,80	6145,23	5471,88	6179,09	4358,14	ca. 7000
Number of cervicals	63+	60	58+	62	63+	72	60	75–76

**Figure 16:**
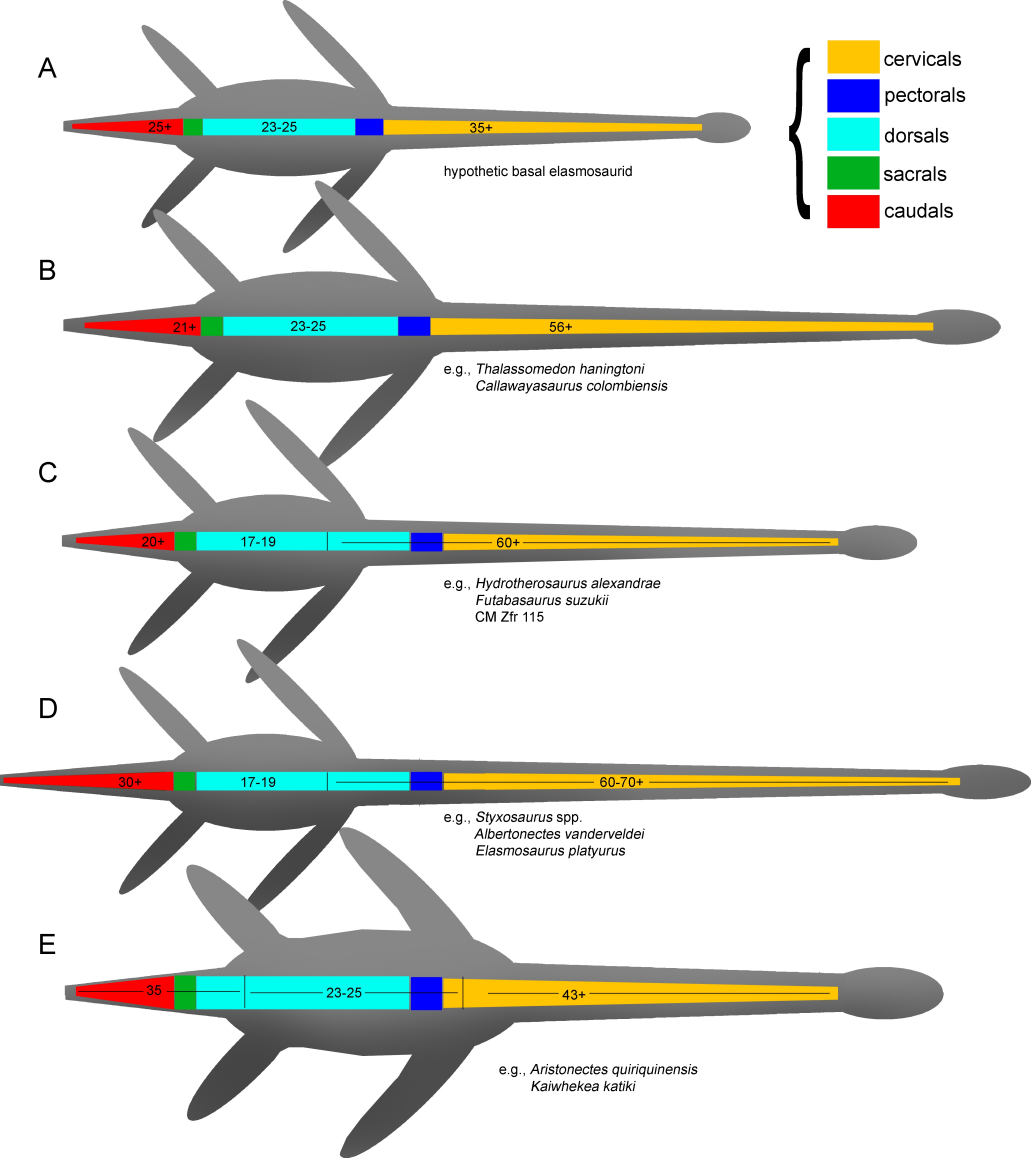
Schematics of the evolution of the elasmosaurid axial skeleton. (A) hypothetic elasmosaurid ancestor with ‘*Cimoliasaurus*’-grade cervicals and likely 23–25 dorsals. (B) ‘Mid’ Cretaceous event(s) of neck elongation by the acquisition of additional cervical centra and retention of plesiomorphic dorsal count, as it occurs on *Thalassomedon haningtoni* and *Callawayasaurus colombiensis*. (C) Santonian-Maastrichtian neck elongation by homeotic shifting of anteriormost dorsals and pectorals into the neck, with a reduction of the dorsal to 17–19 centra, and by acquisition of few additional cervical centra. This seems to be the case of *Futabasaurus suzukii* (the neck is incomplete), *Hydrotherosaurus alexandrae* and CM Zfr 115. (D) Campanian extreme neck elongation event due to the combined effect of the homeotic shifting of anteriormost dorsals and pectorals into the neck, causing a reduction of the dorsal count to 17–19 centra; also coupled to the acquisition of several additional cervical centra, and by the acquisition of extremely elongated individual centra (‘can-shaped’ cervicals). Caudal count is also increased. Examples of this are all the Styxosaurines. (E) Maastrichtian shortening event associated to a homeotic shifting of the trunk, leaving 45 cervicals, 23–24 dorsals and 35 caudals. Centra also become very short and very broad. Examples of this are all the aristonectines. The plesiomorphic dorsal count is recovered, however, the total axial count is remarkably similar to the case (C). Thus, aristonectines are likely derived from elasmosaurids with ‘*Cimoliasaurus*’-grade cervicals.

Based on the axial formula of sister taxa of the Elasmosauridae, an estimated expected formula for an hypothetic basal elasmosaurid can be proposed (Fig. 16A), with 35 or more cervical vertebrae, 23–25 dorsal vertebrae and 25 or more caudal vertebrae. The ‘mid’ Cretaceous *C. colombiensis* and *T. haningtoni* retained such postcervical formula with a minor reduction of the caudal count (at least on *T. haningtoni*; unknown on *C. colombiensis*), however, their necks became larger through the acquisition of additional cervical vertebrae, reaching more than 56 (Fig. 16B), but without acquiring very elongated centra (‘can-shaped’) in some parts of the neck. A second event is represented by Late Cretaceous (Santonian-Maastrichtian) elasmosaurids such *Futabasaurus suzukii* (Japan), *Hydrotherosaurus alexandrae* (California) and CM Zfr 115 (New Zealand). In these forms, a homeotic shifting occurs on pectoral and anterior dorsal vertebrae, which passes onto the neck, increasing the cervical count. The caudal count is mostly retained (Fig. 16C). Another condition, likely derived from the second event (C), is represented by the most extreme neck elongation due to the acquisition of additional cervical vertebrae, but retaining the dorsal count of the event (C). Coupled to this is the acquisition of elongated cervical vertebrae (‘can-shaped’). The caudal count is also increased, giving these forms the most extremely elongated aspect among elasmosaurids (Fig. 16D). Examples of this are all the styxosaurines (i.e., *Styxosaurus* spp., *Elasmosaurus platyurus*, *Albertonectes vanderveldei* and *Terminonatator pointeixensis*). Finally, another event occurred during the Maastrichtian and is represented by a homeotic shifting of the trunk in the anterior direction. This caused a reduction of the cervical count below 45, the increasing of the dorsal count to 23–25 (recovering the ancestral condition) and the increasing of the caudal count to 35. This is the case of the aristonectines (Fig. 16E). This condition can be only verified in *Aristonectes quiriquinensis*, based on the two skeletons available (holotype SGO.PV.957 and referred SGO.PV.260) and partially on *Kaiwhekea katiki*, while other known aristonectines (i.e., *Aristonectes parvidens*, “*Morturneria*” *seymourensis* and *Alexandronectes zealandiensis*) have incomplete postcranial skeletons which encumber the evaluation of such features. Interestingly, the axial count of *Aristonectes quiriquinensis* is estimated on 108 (Otero et al., 2014c), which is remarkably similar to the axial count of *H. alexandrae* and CM Zfr 115 (109, respectively). This evidence suggests that the dorsal count estimated for aristonectines is not the result of an atavism and subsequent expression of the ancestral elasmosaurid condition. Instead, it seems to be a convergence as a consequence of the general anterior shifting of the trunk. It is likely that the aristonectines are a lineage derived from elasmosaurids with ‘*Cimoliasaurus*’-grade cervical vertebrae instead being an old lineage derived from Lower or ‘mid’ Cretaceous elasmosaurids. The latter is supported by the known fossil record of aristonectines, so far restricted to the upper Campanian-Maastrichtian of the Southern Hemisphere.

**Neck length, Cervical Count and Ontogeny**—The neck length of elasmosaurids is highly variable (O’Keefe & Hiller, 2006). Values estimated in Table 7 show that the neck length of *Elasmosaurus platyurus* is almost the same of *Thalassomedon haningtoni*, however, the first possesses 72 cervical vertebrae while 62 are preserved in the latter. This implies that each cervical centrum of *T. haningtoni* is proportionally larger than each cervical centrum of *Elasmosaurus platyurus*, condition that is verified with the absolute measurements used here for the bivariate analysis. Furthermore, the bivariate analysis shows that *T. haningtoni* lacks disparate cervical centra (‘can-shaped’) such as those present in *Elasmosaurus platyurus*, AMNH 1495, AMNH 5835, *Styxosaurus snowii* and *Albertonectes vanderveldei*. All these facts show in numbers that *T. haningtoni* was a systemically bigger animal, with a very long neck, but also with cervical vertebrae comparatively higher and broader than those of *Elasmosaurus platyurus*. Then, as previously noted by O’Keefe & Hiller (2006), the effective neck length is a feature that depends on the cervical count, but also depends on the ontogenetic stage of the animal. Even when comparing adult individuals, differences could appear as a consequence of the continuous growth throughout their life, as it occurs in extant reptiles such as crocodiles or turtles (Romer, 1956). On the other hand, the longest known elasmosaurid neck is that of *Albertonectes vanderveldei*, with ca. 7 m and 76 cervical vertebrae (discussed to be 75 according Sachs, Kear & Everhart, 2013). All these facts evidence that the effective neck length is the combined result of a high cervical count as well as the possession of a large body size on mature or even old ontogenetic stages. Because of this, the comparison of neck length between different individuals cannot be considered a reliable morphologic character with unambiguous taxonomical value.

**Paleobiogeographic and chronostratigraphic distribution**—Integrating the evolution of the axial formula and the pectoral girdle with the biogeographic and chronostratigraphic distribution of each taxon returned consistent patterns. Regarding the North American elasmosaurids from the Western Interior Seaway, a likely first stage of neck elongation is represented by *T. haningtoni* + *L. morgani*. Both have similar ages (Cenomanian and Cenomanian-Turonian, respectively); both taxa also have cervical vertebrae without disparate elements (‘can-shaped’ cervical vertebrae); both taxa possess more than 60 cervical vertebrae, and furthermore, both taxa have pectoral girdles with large scapulae and large, well-ossified claviculae and interclavicular. A second stage within the Western Interior Seaway is represented by the styxosaurines, which acquired the most elongated necks among plesiosaurians. All of them are Campanian, all of them likely have more than 60 cervical vertebrae, and more than 70 in some taxa, and they are also characterized by possessing disparate ‘can-shaped’ mid-cervical vertebrae. Finally, all styxosaurines preserving the pectoral girdle lack a pectoral bar, they possess large scapulae, and likely have reduced or poorly ossified clavicle/interclavicular (Welles, 1943; Welles, 1949).

Lower Cretaceous, subequatorial elasmosaurids are still scarcely known in terms of diversity, however, they are represented by a highly informative taxon, *C. colombiensis*. This taxon possesses axial and pectoral features that appear as an intermediate between Leptocleidia and the ‘mid’ Cretaceous elasmosaurids from the Western Interior Seaway (i.e., *T. haningtoni* and *L. morgani*).

On the other hand, Late Cretaceous elasmosaurids from the Pacific Realm with well-preserved skeletons (e.g., *Hydrotherosaurus alexandrae*, CM Zfr 115) possess necks with more than 60 cervical vertebrae, they are all restricted to the Santonian-Maastrichtian, and they do not possess ‘can-shaped’ cervical vertebrae nor a pectoral bar, while the interclavicular and clavicles seem to be non-ossified or even absent.

Finally, aristonectines are restricted to the upper Campanian-Maastrichtian. They represent a late stage in elasmosaurid evolution. These animals possess disparate cervical vertebrae that are shorter and broader than other elasmosaurids; they also have shortened necks through a reduction of the cervical count, they possess large heads, axially elongated bodies, large extremities and elongated tails. Aristonectines are not closely related to styxosaurines. Each lineage evolved independently. The evidence here presented suggests that both clades evolved from different elasmosaurid ancestors with ‘*Cimoliasaurus*’-grade cervical vertebrae. Prior to the Campanian (time lapse estimated for the divergence of each clade), the latter forms were abundant and widely spread along the Pacific and the Western Interior Seaway, respectively.

**Figure 17:**
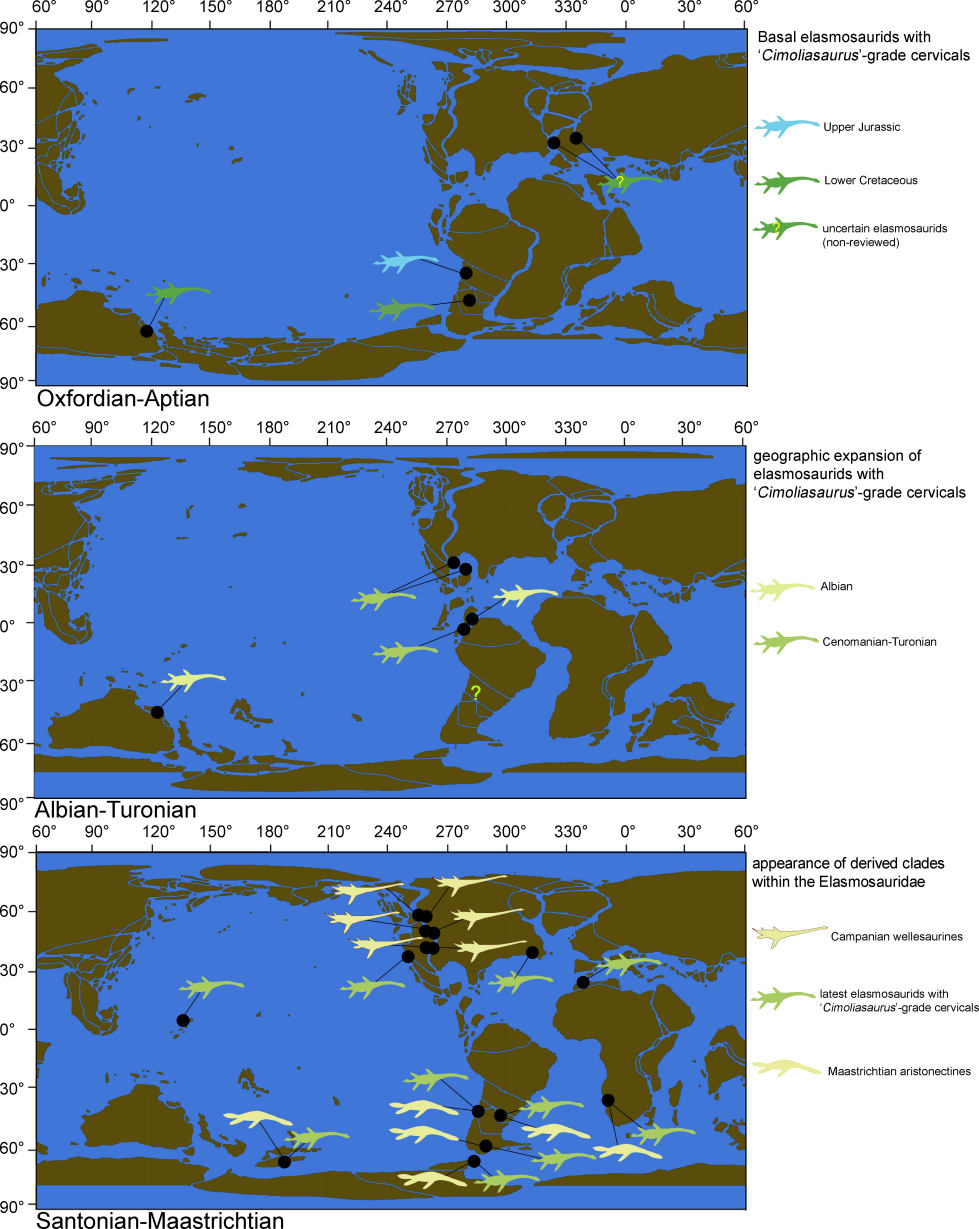
Biogeographic changes of the Elasmosauridae. Maps showing major changes on the distribution of different elasmosaurid morphotypes between the Upper Jurassic-Late Cretaceous.

Chronologically, the earliest elasmosaurid occurrence are likely represented by postcranial material from the Oxfordian of northern Chile (Otero et al., 2015b). Lower Cretaceous records include the Hauterivian ‘Speeton Clay plesiosaurian’ from Yorkshire, U.K. (Benson & Druckenmiller, 2014). South American records include upper Valanginian-lower Hauterivian elasmosaurids with cervical vertebrae referable to the ‘*Cimoliasaurus*’-grade morphotype (O’Gorman et al., 2015b). Cervical vertebrae with the same proportions have been reported from the Aptian of New South Wales, Australia (Kear, 2002). The morphotype persists through the ‘middle’ Cretaceous and is represented by specimens from the upper Albian of Queensland, Australia (Kear, 2001), by *Callawayasaurus colombiensis* from the lower Aptian of Colombia (Welles, 1962), and by the Cenomanian-Turonian, indeterminate elasmosaurid AMNH 6796 (type of ‘*Alzadasaurus tropicus*’ Colbert, 1949). The oldest records within the Western Interior Seaway are represented by two taxa with ‘*Cimoliasaurus*’-grade cervical vertebrae, these being the lower Cenomanian *T. haningtoni* (DMNH 1588) and the Turonian *L. morgani* (SMU SMP 69120) (Welles, 1943; Welles, 1949). During this lapse, records from South America are lacking. During the Santonian-Maastrichtian, elasmosaurids reached a widespread distribution. ‘*Cimoliasaurus*’-grade cervical vertebrae from the north Pacific are represented by *Futabasaurus suzukii* (NSM PV15025) from Japan (Sato, Hasegawa & Manabe, 2006), and by *Hydrotherosaurus alexandrae* (UCMP 33912) from the Maastrichtian of California, USA. Along the south Pacific realm, elasmosaurids with the latter cervical morphotype are well represented by several informative enough specimens: CM Zfr 115 from the upper Campanian of New Zealand (Hiller et al., 2005) and SGO.PV.6506 from the upper Maastrichtian of central Chile (Otero et al., 2014a). Specimens with ‘*Cimoliasaurus*’-grade cervical vertebrae are also known in Antarctica, represented by *Vegasaurus molyi* from the lower Maastrichtian of Vega Island (O’Gorman et al., 2015a). Additional elasmosaurids with the same cervical morphotype also occur in the south Atlantic, represented by SGO.PV.6558 from the upper Maastrichtian of the Magallanes Basin (Otero et al., 2015a), and by MCS PV 4 from the upper Campanian-lower Maastrichtian of Argentinean Patagonia (Gasparini, Salgado & Parras, 2007). North Atlantic occurrences are represented by the type specimen of ‘*Cimoliasaurus magnus*’ from the Maastrichtian of New Jersey (Leidy, 1851). Complementary occurrences are known from the lower Maastrichtian (Araújo et al., 2015b; Araújo et al., 2015a) and upper Maastrichtian of north Africa (Lomax & Wahl, 2013). Finally, the two divergent clades Styxosaurinae and Aristonectinae are restricted to the Campanian of the Western Interior Seaway and the upper Campanian-Maastrichtian of the Weddellian Biogeographic Province, respectively. A general scheme of these occurrences is summarized (Fig. 17).

In this sense, the previous proposal of Otero et al. (2014b; 2015a) on the presence of extremely-long necked elasmosaurids in the upper Campanian of Antarctica and the Maastrichtian southern South America actually represents cervical vertebrae with disparate proportions among elasmosaurids with ‘*Cimoliasaurus*’-grade cervical vertebrae, but these examples do not correspond to cases comparable to the ‘can-shaped’ cervical vertebrae typical of the Western Interior Seaway. Thus, elasmosaurids with extreme necks such as those of the styxosaurines are so far absent in the Southern Hemisphere.

## Conclusions

A revision of the Campanian AMNH 1495 and AMNH 5835 proved that both specimens are closely related and belong to elasmosaurids with extremely long necks. Such forms were exclusively restricted to the Western Interior Seaway of United States and Canada. Additional specimens with similar necks are represented by *Albertonectes vanderveldei*, *Elasmosaurus platyurus*, *Styxosaurus snowii* and *Terminonatator pointeixensis*, all of them being Campanian in age. AMNH 1495 is here concluded to be congeneric with AMNH 5835, but also with *Styxosaurus snowii*, and thus, both specimens are now placed within the genus *Styxosaurus.* Previous referral of AMNH 5835 to *Styxosaurus browni* is here revalidated, while AMNH 1495 is referred to *Styxosaurus* sp. due to the lack of its skull. As a consequence, the former genus *Hydralmosaurus*, previously fixed to AMNH 1495 and having AMHN 5835 as referred specimen, is here considered as a junior synonym of *Styxosaurus.*

Regarding typical Campanian elasmosaurids from the Western Interior Seaway, an unambiguous type of cervical vertebrae is noted to occur in variable positions among the neck. Such vertebrae are here called “can-shaped” cervical morphotype, being almost twice as long as they are high and with similar height and breadth. The ‘can-shaped’ cervical morphotype does not occur in elasmosaurids outside the Western Interior Seaway. Additional support based on phylogenetic and bivariate analyses allows proposing a new clade for these extreme elasmosaurids, here named Styxosaurinae. On the other hand, this research identifies a plesiomorphic cervical type characterized by centra that are about two times broader than high and similarly as high as they are broad. This kind of cervical vertebrae were historically referred as typical of the genus *‘Cimoliasaurus*’ and later considered typical of the clade ‘Cimoliasauridae’ (both taxa currently considered as *nomina dubia*). This study recuperates this morphological concept, coining the ‘*Cimoliasaurus*’-grade cervical morphotype in reference to the first description of those vertebrae in AMNH 2554 (type of ‘*Cimoliasaurus magnus*’ Leidy, 1851, currently *nomen dubium*). With the exception of styxosaurines and aristonectines, the ‘*Cimoliasaurus*’-grade cervical morphotype is the most common condition among elasmosaurids. Furthermore, this type of cervical vertebrae is present along the whole neck in non-aristonectine and non-styxosaurine elasmosaurids, having a low dispersion in their cervical proportions as shown here by means of bivariate analysis. Then, *Elasmosaurus platyurus* (ANSP 10081), the name-bearing specimen of the Elasmosauridae, is actually a highly derived, endemic elasmosaurid of the Western Interior Seaway, and indeed represents an unusual form among elasmosaurids.

During the evolutionary history of Elasmosauridae, neck length suffered changes on different time lapses. This research shows at least three events of neck variation, represented by a ‘mid’ Cretaceous elongation event along the southern Western Interior Seaway and the subequatorial realm linked to an increased cervical number (56 or more), a retention of the plesiomorphic dorsal count (23–25), and the presence of pectoral girdles with pectoral bar and massive interclavicular, claviculae and scapulae. Examples of this are the Aptian *Callawayasaurus colombiensis*, the Cenomanian *Thalassomedon haningtoni* and the Turonian *Libonectes morgani* (also, likely *Wapuskanectes betsynichollsae* but its neck is incomplete). A second event of neck length variation is represented by the styxosaurines of the Western Interior Seaway that acquired the most extreme necks among elasmosaurids through a drastic increasing of the cervical count (60–76), coupled with homeotic shifting of pectoral and anterior dorsal vertebrae (dorsal count falls to 17–19), and also with pectoral girdles with comparatively reduced interclavicular, clavicle and scapulae. Along the Pacific realm, Late Cretaceous elasmosaurids show a trend to reduce the size of the scapulae, the interclavicular and the claviculae, while among latest Pacific elasmosaurids these elements were not even ossified and could be eventually lost. Among the latter group, a third event of neck length variation is represented by the aristonectines which suffered a reverse shifting with cervical vertebrae passing into the trunk (cervical count falls to 43–45), coupled with an increase of the dorsal count (23–25) as well as the caudal count (35). A likely additional length variation event within the Elasmosauridae May have occurred in the case of the Speeton Clay plesiosaurian and closely related forms, albeit at the moment these are poorly known.

## Supplemental Information

10.7717/peerj.1777/supp-1 Supplemental Information 1 Data matrix of Benson & Druckenmiller (2014) with 80 OTUs, plus twelve additional OTUs added in this study This data matrix was used for all the phylogenetic analyses performed on the Supplementary Data.

10.7717/peerj.1777/supp-2 Data S1 Supplementary Data Different phylogenetic analyses performed for testing the stability of the Styxosaurinae.

## Institutional Abbreviations

AMNH: American Museum of Natural History, New York, United States
AM: Australian Museum, Sydney, Australia
ANSP: Academy of Natural Sciences of Philadelphia, United States
BRSMG: Bristol City Museum and Art Gallery, Bristol, UK
CM Zfr: Canterbury Museum, Christchurch, New Zealand
DMNH: Denver Museum of Natural History, Colorado, United States
GNS: Geology National Survey, New Zealand
GWWU: Geomuseum der Westfaelischen Wilhelms-Universität, Münster, Germany
KUVP: Kansas University, Vertebrate Paleontology, United States
MIWG: ‘Dinosaur Isle’ Museum of Isle of Wight Geology, Sandown, UK
MNA V: Vertebrate Paleontology Collection, Museum of Northern Arizona, USA
MLP: Museo de La Plata, Buenos Aires, Argentina
MOZ.PV: Museo Juan Olsacher, Paleontología de Vertebrados, Zapala, Neuquén, Argentina
MUHNCAL: Museo de Historia Natural y Cultural del Desierto de Atacama, Calama, Chile
NSM: National Science Museum, Tokyo, Japan
OU: Otago University, Dunedin, New Zealand
QM: Queensland Museum, Brisbane, Australia
RSM: Royal Saskatchewan Museum, Canada
SDSM: South Dakota School of Mines, Rapid City, USA
SGO.PV: Museo Nacional de Historia Natural, Paleontología de Vertebrados, Santiago, Chile
SMUSMP: Southern Methodist University, Shuler Museum of Paleontology, Dallas, USA
TPM: Tyrrell Museum of Palaeontology, Alberta, Canada. TTU, Texas Tech University, United States
UCMP: University of California, Museum of Paleontology, United States
USNM: Univesity of Nebraska State Museum, Lincoln, USA
YPM: Yale Peabody Museum, USA

## Additional Information and Declarations

### Data Availability

The following information was supplied regarding data availability: The raw data has been supplied as a Data S1.

### New Species Registration

The following information was supplied regarding the registration of a newly described species: New Taxon LSID urn:lsid:zoobank.org:act:A4AC6098-5485-4FE6-86AA-E368F18CE8DB Publication LSID urn:lsid:zoobank.org:pub:1E0DA2E1-50A9-453D-BC3F-D036DA44B389.
