# Supplementary material for: Taxonomic reassessment of Hydralmosaurus as Styxosaurus: new insights on the elasmosaurid neck evolution throughout the Cretaceous
Source: PeerJ. 2016 Mar 15;4:e1777. doi: 10.7717/peerj.1777 (PMC4806632; doi:10.7717/peerj.1777)
Supplement: Data S1 — Different phylogenetic analyses performed for testing the stability of the Styxosaurinae. [file peerj-04-1777-s002.pdf]

SUPPLEMENTARY DATA: Pylogenetic analysis.

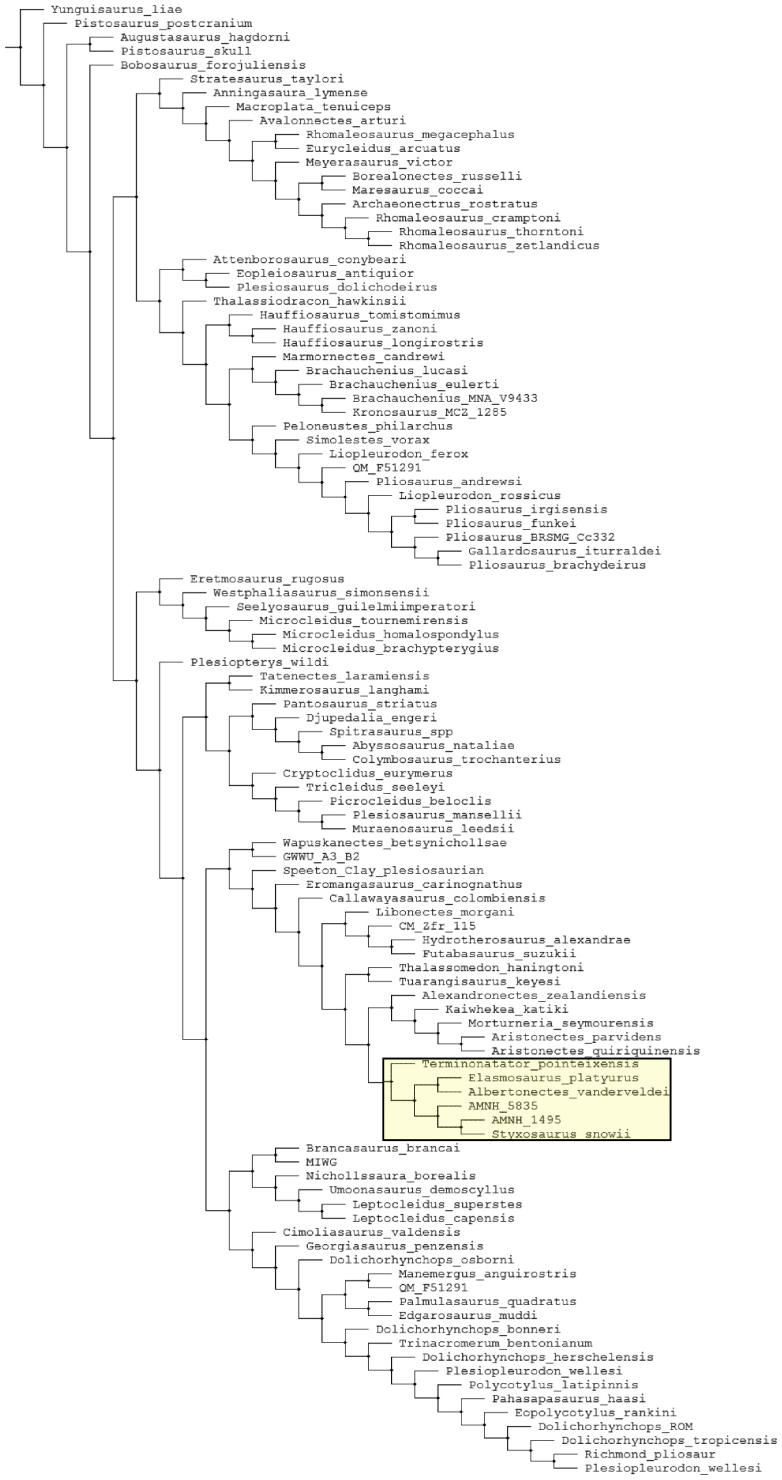

Figure 1: Single most parsimonious cladogram (CI=0.27; RI=0.68; 2511 steps) obtained with implied weighting (K=3; New Technology Search, Tree Fusing, random seed=1) from the datamatrix of Benson and Druckenmiller (2014) plus thirteen elasmosaurid taxa added in this study (105 OTU's). The clade Styxosaurinae is indicated in the highlighted block.

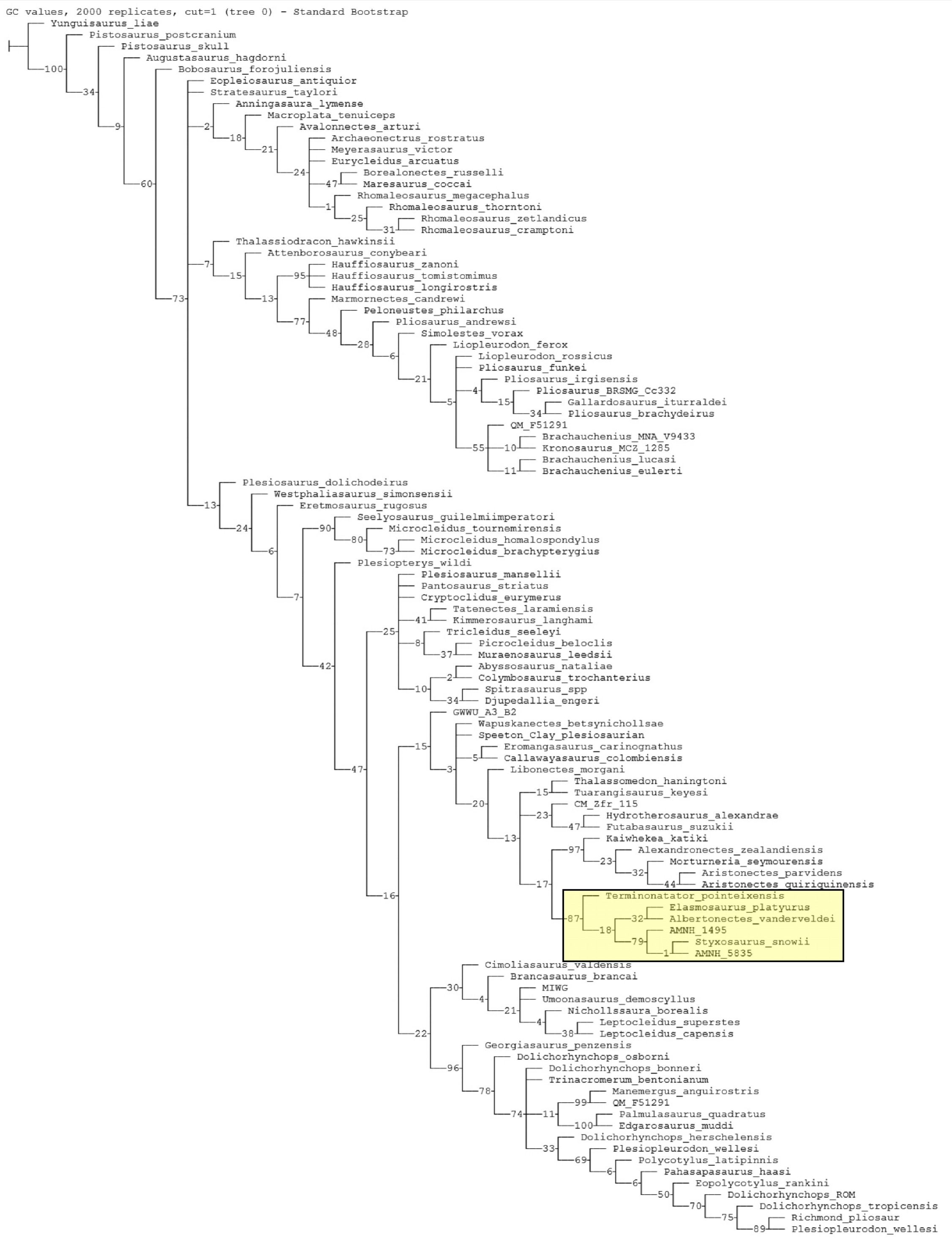

Figure 2: Cladogram obtained with the datamatrix of Benson & Druckenmiller (2014) plus thirteen elasmosaurid taxa added in this study, applying Bootstrap with 2,000 replicates (Standard, New Tech Search, Tree fusing). Good stability (87%) was returned for the Styxosaurinae (highlighted block). The congenerity of AMNH 5835, AMNH 1495 and *Styxosaurus snowii* has good support (79%). The clade Elasmosauridae was returned as unstable (15%).

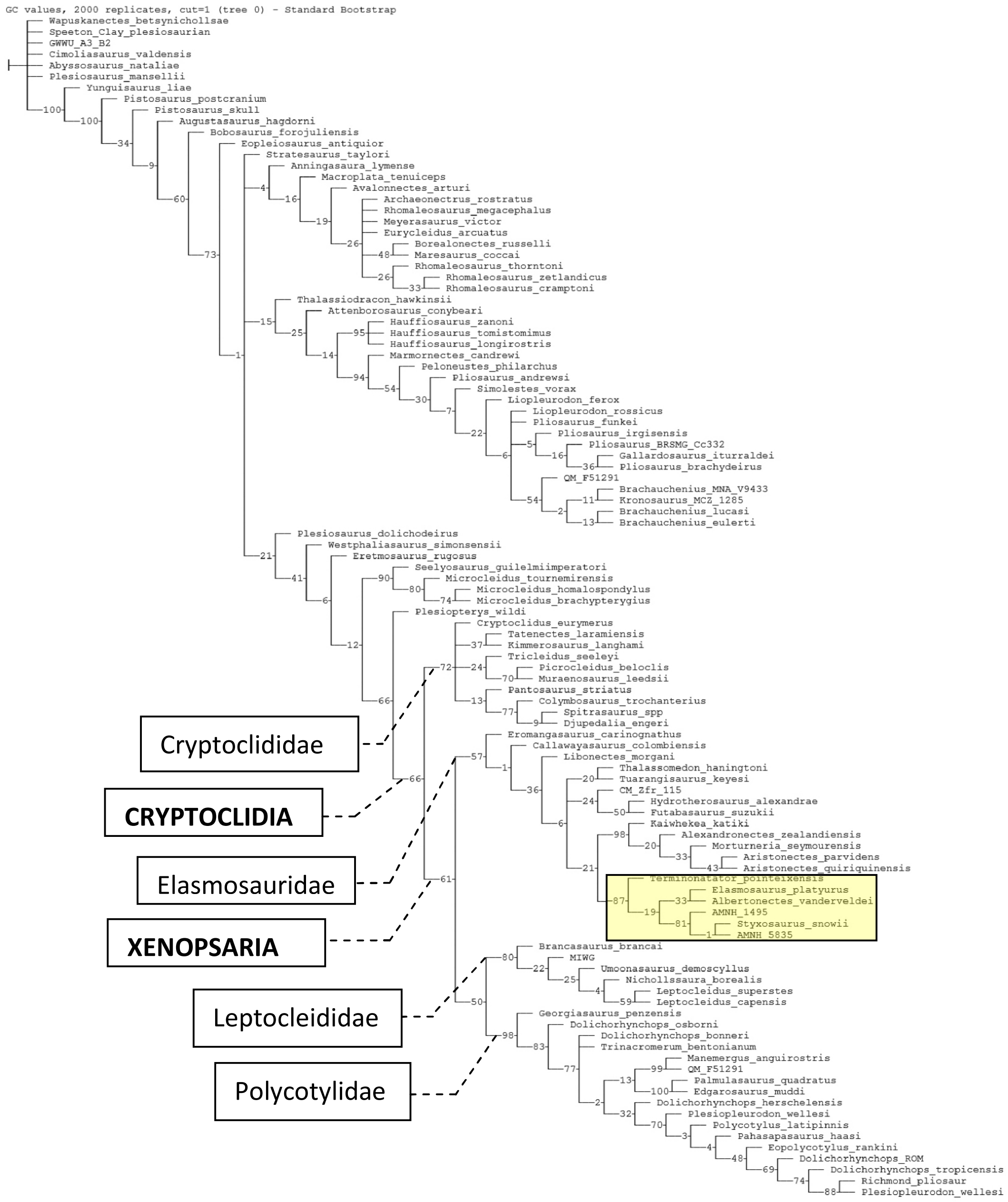

Figure 3: Cladogram obtained applying Bootstrap with 2,000 replicates (Standard, New Tech Search, Tree fusing) to the datamatrix of Benson & Druckenmiller (2014) plus thirteen elasmosaurid taxa added in this study. Pruned taxa include *Wapuskanectes betsynochollsae*, the leptocleidid *Gronausaurus wegneri* (obtained as an elasmosaurid in Benson & Druckenmiller, 2014), as well as '*Cimoliasaurus*' *valdensis*, '*Plesiosaurus*' *mansellii*, *Abyssosaurus nataliae* and Speeton Clay Plesiosaurian. The clades Cryptoclidia and Xenopsaria were returned with good stability (66% and 61%, respectively). Family-level clades within the Cryptoclidia have better stability: Xenopsaria=61%; Elasmosauridae=57%; Leptocleididae=80%; Polycotylidae; 98%; and Cryptoclididae=72%. The clade Styxosaurinae (highlighted block) is well supported (87%). Congenerity of AMNH 5835, AMNH 1495 and *Styxosaurus snowii* is also well supported (81%).
